# Ordered Oxygen Vacancies in the Lithium-Rich Oxide Li_4_CuSbO_5.5_, a Triclinic Structure Type Derived from the Cubic Rocksalt Structure

**DOI:** 10.1021/acs.inorgchem.1c02882

**Published:** 2021-12-06

**Authors:** Arnaud J. Perez, Andrij Vasylenko, T. Wesley Surta, Hongjun Niu, Luke M. Daniels, Laurence J. Hardwick, Matthew S. Dyer, John B. Claridge, Matthew J. Rosseinsky

**Affiliations:** † Department of Chemistry, University of Liverpool, Crown Street, Liverpool L69 7ZD, United Kingdom; ‡ Stephenson Institute for Renewable Energy, University of Liverpool, Chadwick Building, Peach Street, Liverpool L69 7ZF, United Kingdom

## Abstract

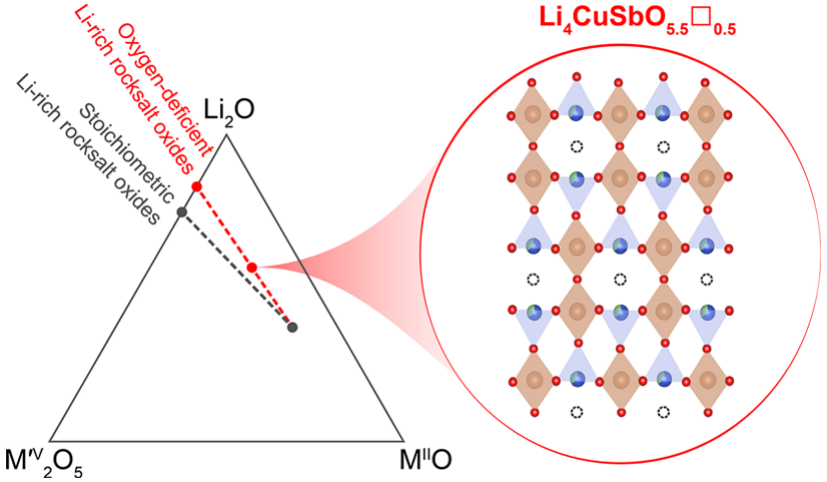

Li-rich rocksalt oxides are promising candidates as high-energy density cathode materials for next-generation Li-ion batteries because they present extremely diverse structures and compositions. Most reported materials in this family contain as many cations as anions, a characteristic of the ideal cubic closed-packed rocksalt composition. In this work, a new rocksalt-derived structure type is stabilized by selecting divalent Cu and pentavalent Sb cations to favor the formation of oxygen vacancies during synthesis. The structure and composition of the oxygen-deficient Li_4_CuSbO_5.5_□_0.5_ phase is characterized by combining X-ray and neutron diffraction, ICP-OES, XAS, and magnetometry measurements. The ordering of cations and oxygen vacancies is discussed in comparison with the related Li_2_CuO_2_□_1_ and Li_5_SbO_5_□_1_ phases. The electrochemical properties of this material are presented, with only 0.55 Li^+^ extracted upon oxidation, corresponding to a limited utilization of cationic and/or anionic redox, whereas more than 2 Li^+^ ions can be reversibly inserted upon reduction to 1 V vs Li^+^/Li, a large capacity attributed to a conversion reaction and the reduction of Cu^2+^ to Cu^0^. Control of the formation of oxygen vacancies in Li-rich rocksalt oxides by selecting appropriate cations and synthesis conditions affords a new route for tuning the electrochemical properties of cathode materials for Li-ion batteries. Furthermore, the development of material models of the required level of detail to predict phase diagrams and electrochemical properties, including oxygen release in Li-rich rocksalt oxides, still relies on the accurate prediction of crystal structures. Experimental identification of new accessible structure types stabilized by oxygen vacancies represents a valuable step forward in the development of predictive models.

## Abstract 2

Controlling the composition and synthesis conditions of Li-rich rocksalt oxides can lead to to new structure types stabilized by oxygen vacancies. The complex atomic ordering of Li_4_CuSbO_5.5_ is described and discussed in the context of other oxygen-deficient rocksalt oxides.

## 1 Introduction

The development of high-energy density cathode materials for Li-ion batteries has opened a large field of investigation for solid-state chemists to propose new materials and new energy storage concepts. The discovery of the reversible redox activity of oxygen anions in the so-called “Li-rich” rocksalt oxides with formula Li_1+*x*_M_1–*x*_O_2_ (M = transition metal) represents an important paradigm shift in that field.^1,2^ The possibility to extract electrons from oxygen in addition to the transition metals gives rise to a large increase in the specific capacity of these materials compared with stoichiometric LiMO_2_ layered oxides such as LiCoO_2_ or the NMC phases Li(Ni_1–*x*–*y*_Mn_*x*_Co_*y*_)O_2_. However, this increase in capacity is often accompanied by several shortcomings linked to stabilization mechanisms for oxide anions upon their oxidation. Common issues range from voltage hysteresis between charge and discharge, resulting in a low-energy efficiency, to cation migration and trapping in tetrahedral sites, leading to voltage decay, and irreversible evolution of oxygen from the surface and/or the bulk of the material that causes irreversible capacity loss.^1^ Research efforts are therefore focused on understanding how the redox activity of the anion sublattice can be controlled and finely tuned through appropriate choice of cations, including redox active transition metals and redox inactive elements (alkali, alkaline earth, early transition metals with no d electrons or p-block elements).

The release of gaseous oxygen is a particularly important problem, as it results in irreversible capacity loss and penalizing structural modifications such as densification of the surface of materials^3−5^ or complete amorphization in the most extreme cases.^6^ Computational evaluation of the propensity for a specific composition to undergo oxygen release and oxygen vacancy formation represents a promising path to guide future experimental exploration of high-energy density cathode materials. Several research groups have already taken that direction,^2,7,8^ reporting different indicators to predict the release of oxygen and leading to a more general understanding of the reversibility of oxygen redox activity. Nevertheless, these indicators rely on the accurate prediction of the structure formed upon the introduction of an oxygen vacancy and would benefit from the knowledge of experimentally determined crystal structures adopted by Li-rich rocksalt oxides containing oxygen vacancies.

In this work, the formation of structures with oxygen vacancies is taken as an opportunity to discover new structure types related to the Li-rich rocksalt oxides, with formula Li_4_MM′O_6–*x*_□_*x*_, where M and M′ cations must be selected in order to favor the formation of oxygen vacancies during synthesis rather than during electrochemical oxidation.

Work from McCalla and co-workers on Li–Fe–Sb–O and Li–Ni–Sb–O compounds suggests that Sb-based materials are prone to oxygen release from the bulk or the surface of the material, together with cation reduction upon deep oxidation.^9,10^ This mechanism of reductive elimination is an extreme and irreversible manifestation of the reductive coupling mechanism observed in some cathode materials.^2^ To achieve this reaction at the synthesis step, we further replaced Ni/Fe with Cu, which is unlikely to oxidize beyond divalent Cu^2+^ under normal solid-state synthesis conditions (high temperature, ambient atmosphere). Therefore, we targeted the composition Li_4_CuSbO_5.5_□_0.5_ and report its structure and electrochemical properties. In this new compound, the sum of M and M′ cation oxidation states amounts to 7 instead of 8, as is usually considered to prepare stoichiometric Li_4_MM′O_6_ rocksalt oxides.

## 2 Results

### 2.1 Synthesis and Structure Determination

**Figure 1:**
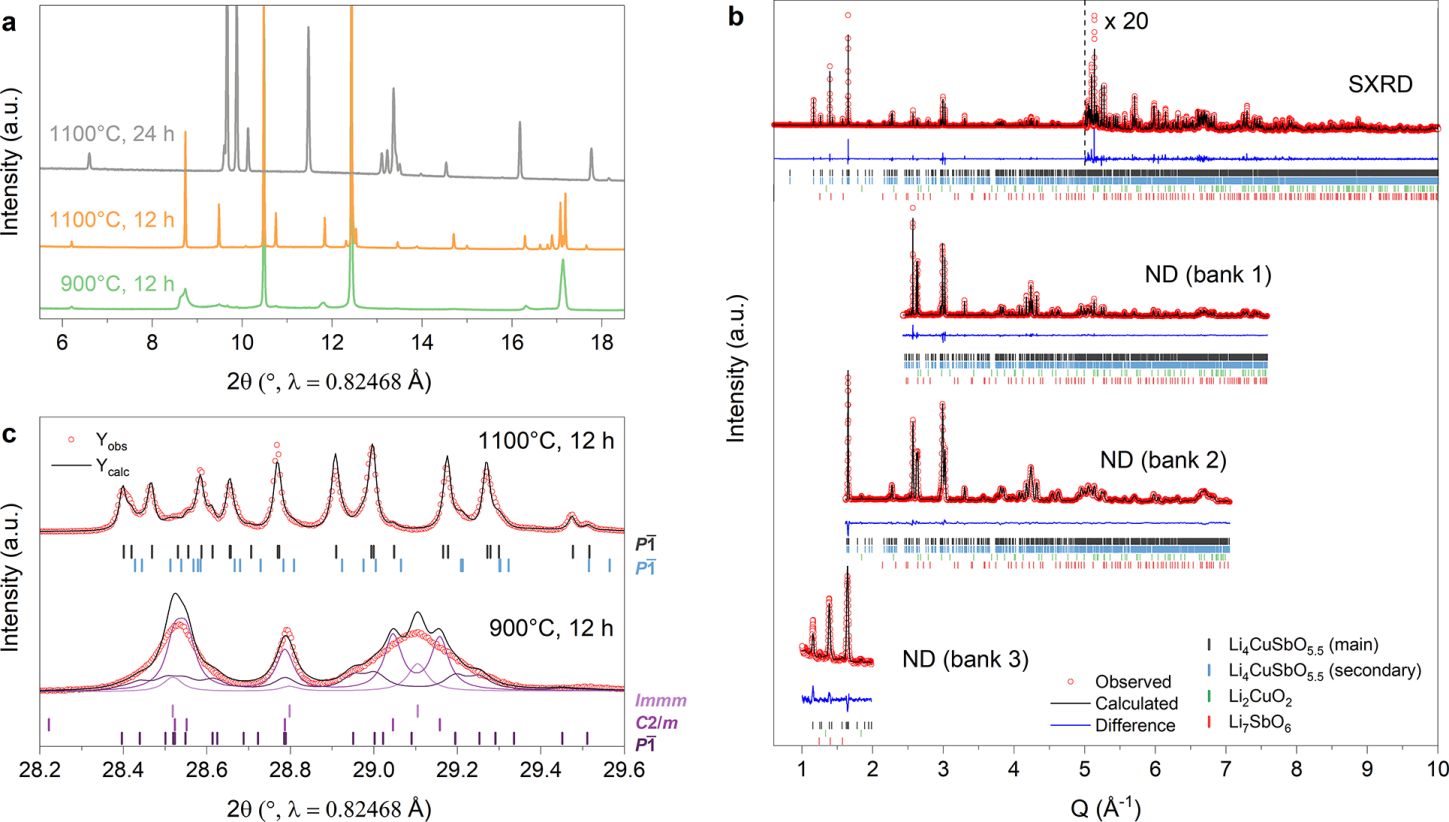
Diffraction data on Li_4_CuSbO_5.5_. (a) XRD patterns of samples prepared at 900 °C for 12 h (green), 1100 °C for 12 h (orange), and 1100 °C for 24 h (gray). The latter corresponds to the formation of the Li_2_O-deficient phase Li_3_CuSbO_5_. (b) Combined Rietveld refinement of neutron and SXRD data for Li_4_CuSbO_5.5_ prepared at 1100 °C for 12 h. Experimental points are marked by red circles, simulated pattern by a black line, difference pattern by a blue line, and reflection position for the main and secondary Li_4_CuSbO_5.5_ phases is in black and blue, and for Li_2_CuO_2_ and Li_7_SbO_6_, impurities are in green and red, respectively. (c) The sample prepared at 1100 °C for 12 h can be reasonably well fitted with two triclinic phases with slightly different cell parameters, whereas the sample prepared at 900 °C for 12 h appears as a mixture of triclinic, monoclinic, and orthorhombic phases isostructural to the parent Li_5_SbO_5_ (*C*2/*m*) and Li_2_CuO_2_ (*Immm*) phases.

Li_4_CuSbO_5.5_ can be prepared by a simple ceramic method starting from Li_2_CO_3_, CuO and Sb_2_O_3_ in proportions Li:Cu:Sb = 4.4:1:1 and heating the hand-ground mixture at 1100 °C for 12 h under air (Figure 1a, orange). Heating at a lower temperature (900 °C) also leads to the formation of Li_4_CuSbO_5.5_ with broader Bragg reflections that indicate lower crystallinity and a less ordered structure (Figure 1a, green). Prolonged heating at 1100 °C (24 h) or higher temperatures results in Li_2_O loss and the decomposition of Li_4_CuSbO_5.5_ into the reported Li_3_CuSbO_5_ phase (Figure 1a, gray).^11^ The 10 mol % excess of Li_2_CO_3_ is therefore important to delay this decomposition while heating at a temperature high enough to obtain a well crystallized material. The final cation stoichiometry was confirmed by inductively coupled plasma optical emission spectroscopy (ICP-OES) to be Li_4.22(9)_Cu_1.027(18)_Sb_1.000(2)_, with deviations from the ideal stoichiometry that can be explained by residual Li_2_CO_3_/Li_2_O, Li_2_CuO_2_ and Li_7_SbO_6_ impurities (<1 wt % according to Rietveld quantitative analysis).^12^

**Figure 2:**
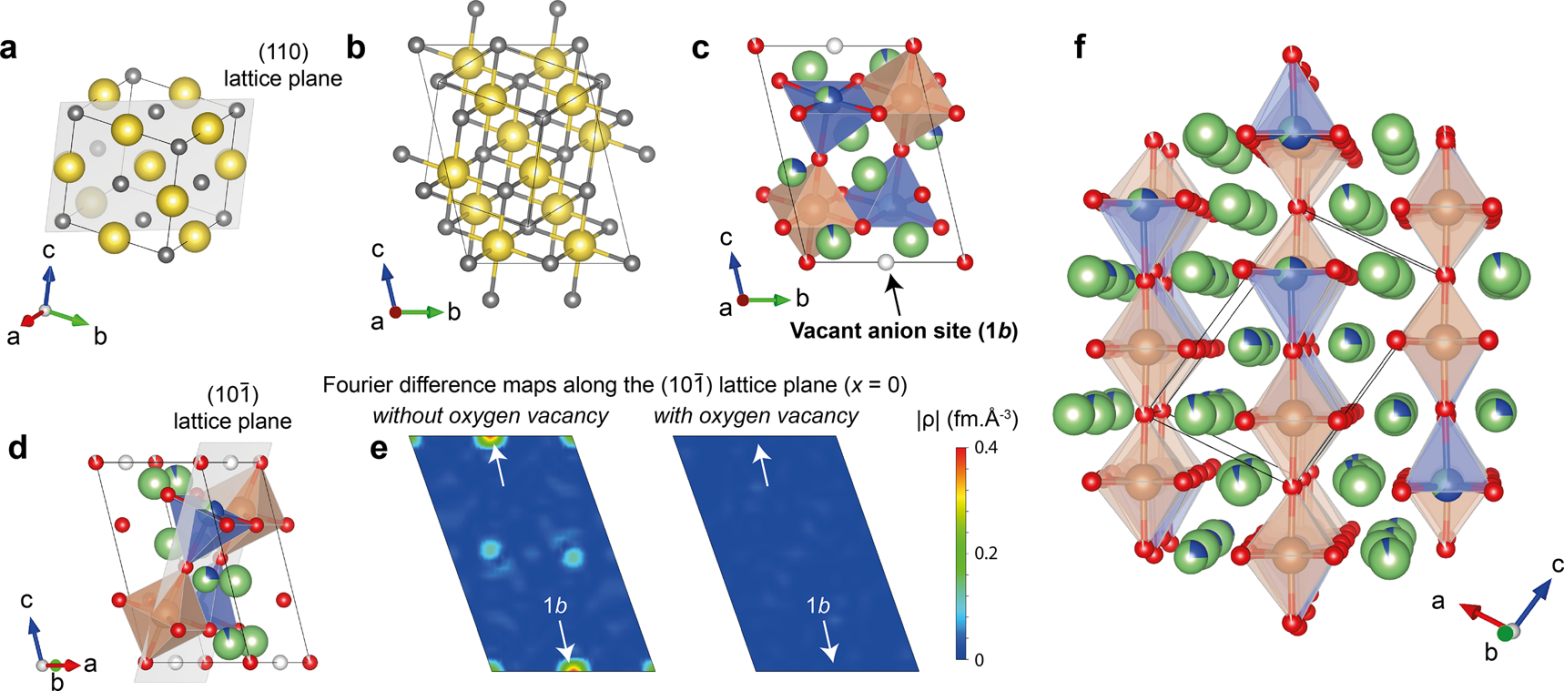
Structure of Li_4_CuSbO_5.5_. The atomic ordering is derived from the NaCl rocksalt structure, represented here in the cubic cell setting (a) and in the triclinic setting (b) corresponding to the structure of Li_4_CuSbO_5.5_ (c). Sodium and chlorine atoms are in yellow and gray, respectively. Lithium, copper, antimony, oxygen atoms, and oxygen vacancies are in green, blue, brown, red, and white, respectively. The presence of oxygen vacancies ordered in the (101) lattice plane (d), corresponding to the (110) lattice plane of the parent cubic structure (a), is confirmed by Fourier difference maps using synchrotron and neutron diffraction data. Nuclear density maps along the (101) lattice plane using the ND bank 1 are compared with and without the presence of oxygen in site 1*b* (e). Site 1*b* is highlighted by white arrows. (f) Perspective view of the structure, with polyhedra shown only around copper and antimony atoms in the (101) lattice planes containing the antimony atoms and oxygen vacancies.

The unit cell was determined by indexing the powder X-ray diffraction (XRD) pattern of the sample prepared at 1100 °C for 12 h using synchrotron radiation. The structure can be indexed using a triclinic cell with cell parameters: *a* = 5.207365(8) Å, *b* = 5.817536(8) Å, *c* = 7.888147(13) Å, α = 100.58195(11)°, β = 96.93693(11)°, and γ = 106.96091(9)°. All attempts to index the pattern with a higher symmetry cell were unsuccessful. Initial positions for Sb, Cu, and O atoms were found by simulated annealing using the program FOX^13^ and further determined through Fourier difference maps as implemented in the FullProf suite.^14^ Li positions were determined from Fourier difference maps using neutron diffraction (ND) data. Overall site occupancies and atomic displacement parameters were determined by a Rietveld refinement combining synchrotron X-ray diffraction (SXRD) data with neutron diffraction data (Figure 1b). The combined refinement with neutron data confirmed the presence of oxygen vacancies in the structure. The occupancies of all oxygen sites were refined freely and were fixed at 1 when reaching values larger than unity during the refinement. Partial occupation of cation sites by Sb, Cu, and Li was refined incrementally, using electronic and nuclear density Fourier difference maps generated by the program GFourier as implemented in Fullprof. Specifically, difference Fourier maps plotted along the (101) lattice plane (slice shown at *x* = 0) clearly support the absence of oxygen in site 1*b* (Figure 2e for ND bank 1). The fit statistics of all banks improve with a global weighted value of χ^2^ decreasing from 45.5 without vacancy to 16.1 with a vacancy. Unsatisfactory fit of the peak shapes, with *hkl*-dependent asymmetries, indicate the presence of inhomogeneous cell parameters in the sample. Adding a secondary Li_4_CuSbO_5.5_ phase to the refinement with the same atomic parameters and slightly different cell parameters (*a* = 5.20263(3) Å, *b* = 5.82002(4) Å, *c* = 7.88000(6) Å, α = 100.4694(6)°, β = 97.0145(5)°, γ = 107.0248(6)°) improved the fit significantly (from χ^2^ = 26.5 with a single phase to 16.1 with two phases, see Figure 1c, top) but still did not achieve a perfect fit of the peak shapes. A distribution of phases with continuously varying cell parameters would likely give a result more representative of the reality. The secondary Li_4_CuSbO_5.5_ phase amount varies between 20 and 32 wt %, depending on the diffraction bank considered, and its volume is 0.17% smaller than that of the dominant phase. It is likely that this secondary phase corresponds to a slightly different composition or atomic ordering compared to the main one. Attempts at refining the composition of this phase led to unstable results, and we therefore decided to keep the same atomic parameters for the main and secondary phases as an approximation. Additional experiments show that cell parameters, and potentially the atomic ordering, are affected by the sample’s cooling conditions (see Methods section). Small additional peaks corresponding to Li_2_CuO_2_ and Li_7_SbO_6_ impurities were also observed in the synchrotron X-ray and neutron data and were added to the fit. The result of the combined refinement is shown in Figure 1b and Table 1, and the structure is presented in Figure 2.

**Table 1:** Structural Parameters for the Main Li_4_CuSbO_5.5_ Phase from a Combined Rietveld Refinement on Neutron and Synchrotron X-ray Diffraction Data

refinement parameters				
formula	Li_8_Cu_2_Sb_2_O_11_			
temperature (K)	298			
pressure	atmospheric			
source	neutron time-of-flight			synchrotron X-ray
data bank	ND, bank 1	ND, bank 2	ND, bank 3	SXRD
angle (°)/wavelength (Å)	168.657	90.248	29.930	0.82468(1)
*d* spacing range (Å)	0.68–2.59	0.89–3.89	3.14–6.28	0.58–9.44
TOF (μs)/2θ (°) range	40000–125000	31000–135500	40000–80000	5–120
TOF (μs)/2θ (°) step	20.3307	48.7172	75.1060	0.004
no. of reflections	1459	1120	15	6082
no. of refined parameters	95			
*R*_p_	13.4	8.78	25.7	14.0
*R*_wp_	11.9	6.89	17.2	16.7
*R*_exp_	5.35	1.96	9.42	3.39
χ^2^	4.92	12.4	3.34	24.3
ρ_min/max_ residuals (fm·Å^–3^/e^–^·Å^–3^)	[−0.004/+0.005]	[−0.003/+0.002]	[−0.0002/+0.0003]	[−0.9/+1.5]
Phases	Estimated Mass Fraction (%)			
Li_4_CuSbO_5.5_, main	79.3	66.2	94.0	76.5
Li_4_CuSbO_5.5_, secondary	20.0	32.3	–	22.1
Li_2_CuO_2_	0.6	0.8	0.9	1.1
Li_7_SbO_6_	0.1	0.7	5.1	0.3

structure parameters for Li_4_CuSbO_5.5_							
space group		*Z*		density (g·cm^–3^)		formula weight (g·mol^–1^)	
*P*1		1		4.459		602.13	
	*a* (Å)	*b* (Å)	*c* (Å)	α (°)	β (°)	γ (°)	volume (Å^3^)
main	5.207365(8)	5.817536(8)	7.888147(13)	100.58195(11)	96.93693(11)	106.96091(9)	220.7860(6)
secondary	5.20263(3)	5.82002(4)	7.88000(6)	100.4694(6)	97.0145(5)	107.0248(6)	220.413(3)

atom	site	*x*	*y*	*z*	occupancy	*B*_iso_ (Å^2^)
O1	2*i*	0.4547(10)	0.4047(9)	0.1628(7)	1	0.66(10)
O2	2*i*	0.5547(9)	0.0990(9)	0.8389(6)	1	0.59(10)
O3	2*i*	0.0067(10)	0.1693(9)	0.6879(7)	1	0.65(11)
O4	2*i*	0.0050(10)	0.3225(8)	0.3134(6)	1	0.35(9)
O5	2*i*	0.4768(10)	0.2336(9)	0.4789(6)	0.959(8)	0.78(11)
O6	1*g*	0	0	0	0.906(6)	0.28(15)
vacancy	1*b*	0	0.5	0	0	–
Sb8	2*i*	0.23390(15)	0.11964(15)	0.24415(10)	1	0.351(4)
Cu9/Li9	2*i*	0.2395(4)	0.6223(4)	0.2526(3)	0.633/0.367(2)	0.35(3)
Cu10/Li10	2*i*	0.7483(8)	0.0411(7)	0.4149(5)	0.249/0.751(2)	0.38(6)
Cu11/Li11	2*i*	0.2691(19)	0.8018(17)	0.9062(13)	0.061/0.939(2)	0.76(19)
Li12	2*i*	0.669(3)	0.691(2)	0.1043(19)	1	2.1(3)
Li13	2*i*	0.749(3)	0.528(3)	0.4070(19)	1	1.3(3)

### 2.2 Structure Description

**Figure 3:**
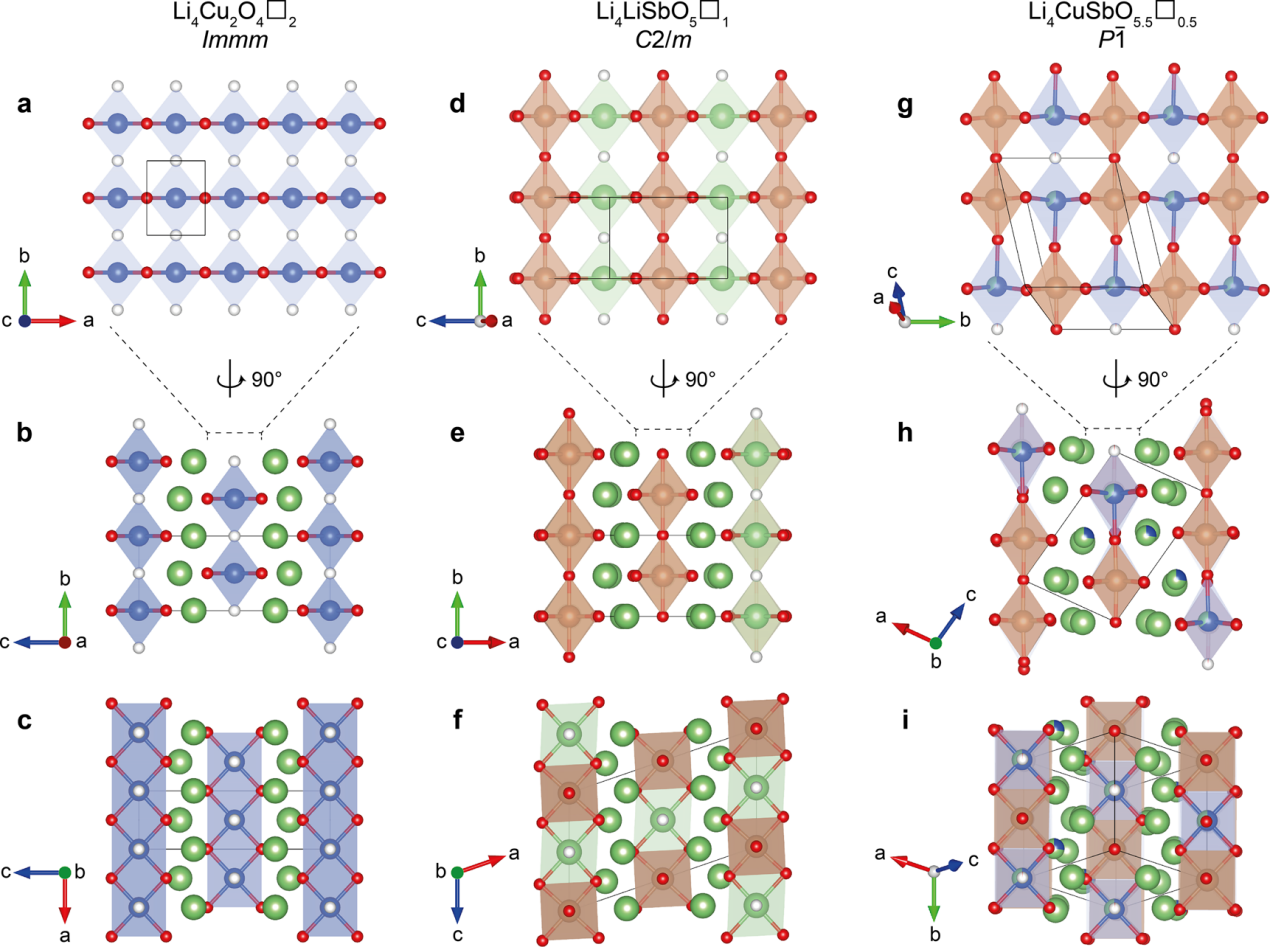
Comparison of rocksalt-derived Li_4_(MM′)O_6–*x*_□_*x*_ crystal structures with oxygen vacancies of Li_2_CuO_2_ (left), Li_5_SbO_5_ (middle), and Li_4_CuSbO_5.5_ (right). Oxygen atoms and oxygen vacancies are represented in red and white, respectively. Copper, antimony, and lithium atoms are in blue, brown, and green, respectively. Polyhedra are shown for M and M′ sites only, including the position of the oxygen vacancies to facilitate the comparison between structures. The top view (a, d, g) represents the (MM′) layers along the (110) lattice plane of the parent cubic structure that clearly displays the ordering of oxygen vacancies with M and M′ atoms. The center view (b, e, h) shows the arrangement of the (MM′) layers separated by Li atoms along the edge-sharing direction, and the bottom view (c, f, i) shows the same arrangement along the corner-sharing direction of M and M′ polyhedra.

The structure of Li_4_CuSbO_5.5_ is derived from the cubic rocksalt structure of NaCl (Figure 2a–c). The unit cell parameters of the triclinic cell are related to those of the cubic cell by the following relation:
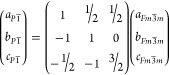
The symmetry lowering from a cubic to a triclinic space group is a consequence of the complex atomic ordering established in Li_4_CuSbO_5.5_. This specific ordering of cations and oxygen vacancies is the result of a balance between competing electrostatic interactions and electronic structure effects. The main features of this arrangement can be most simply understood by focusing on the atomic ordering along the (101) lattice plane (Figure 2d), which corresponds to the (110) lattice plane of the parent cubic structure (Figure 2a). This plane contains the antimony site (Sb8), the main copper site (Cu9/Li9), oxygen sites (O5 and O6), and the vacant oxygen site (site 1*b*). The cation ordering is closely related to those of the parent compounds Li_2_CuO_2_ (also written as Li_4_Cu_2_O_4_□_2_) and Li_5_SbO_5_ (or Li_4_LiSbO_5_□_1_), as shown in Figure 3. All are derived from the cubic rocksalt structure, but the atomic ordering depends on the cationic nature and the content of oxygen vacancies. This can be better understood by writing their formula as Li_4_(MM′)O_6–*x*_□_*x*_: Li_4_(CuCu)O_4_□_2_, Li_4_(LiSb)O_5_□_1_ and Li_4_(CuSb)O_5.5_□_0.5_ with decreasing vacancy content, respectively. The number of oxygen vacancies *x* is directly related to the sum of oxidation states *m* and *m*′ of the cations M and M′ (*x* = (8 – *m* – *m*′)/2).

In the three structures, M and M′ sites are assembled in one plane (Figure 3a,d,g), corresponding to the equivalent (110) lattice plane in the parent cubic rocksalt structure, the (001) plane in the orthorhombic *Immm* cell of Li_2_CuO_2_, the (100) plane in the monoclinic *C*2/*m* cell of Li_5_SbO_5_, and the (101) plane in the triclinic cell of Li_4_CuSbO_5.5_. The M and M′ sites share edges along one direction ([100] for Li_2_CuO_2_, [001] for Li_5_SbO_5_ and [010] for Li_4_CuSbO_5.5_) and corners in the perpendicular direction ([010] for Li_2_CuO_2_ and Li_5_SbO_5_, [212] for Li_4_CuSbO_5.5_). Layers of Li atoms separate each (MM′) layer from the next one (Figure 3b,c,e,f,h,i). The edge-sharing oxygen sites are all fully occupied, whereas the corner-sharing oxygen sites may or may not be occupied depending on the two cations present in the two adjacent M and M′ sites. In the case of Li_4_(CuCu)O_4_□_2_, all M and M′ sites are occupied by Cu^2+^, so that the oxygen corner-sharing sites remain empty, resulting in a square planar coordination for all Cu atoms (Figure 3a,b). Cu sites are therefore connected only by edges, forming ribbons along the [100] direction (Figure 3a). The electronic configuration of Cu^2+^ favors strong Jahn–Teller distortion of its coordination environment, making this square planar environment relatively stable. In Li_4_(LiSb)O_5_□_1_, the high valence of Sb^5+^ cations prevents oxygen vacancies from forming in their immediate surrounding, preserving the octahedral coordination environment. Instead, oxygen vacancies are found between Li^+^ ions, which are, therefore, sitting in a square planar environment. Sb sites form chains of corner-shared octahedra along the [010] direction, and each chain is separated from the next one by square planar Li sites in the [001] direction (Figure 3d). Turning back to Li_4_(CuSb)O_5.5_□_0.5_, we now understand that oxygen vacancies will preferentially be found in corner-shared sites between two Cu^2+^ cations, rather than at a vertex of the octahedral Sb^5+^ cation site. This is possible by ordering Cu^2+^ and Sb^5+^ cations in the (101) plane to alternate between Cu and Sb along the edge-sharing [010] direction −(Cu–Sb–Cu–Sb)– and between pairs of Cu and Sb along the corner shared [212] direction −(Cu–Cu–Sb–Sb)– (Figure 3g). Oxygen vacancies are therefore found between two Cu^2+^ cations, each sitting in opposingly oriented square pyramidal sites. The Cu–O distance at the apex of the pyramid (*d*(Cu9–O5) = 2.288(5) Å) is significantly longer than the Cu–O equatorial distances forming the base of the pyramid (1.975(6)–2.045(7) Å) due to the strong Jahn–Teller distortion discussed above (Figure S1 and Table S1 in SI). The Sb^5+^ cations reside in pairs of octahedral sites connected by a corner. Generally, the repulsion between highly charged cations in rocksalt oxides is the main driving force for cation ordering, leading to completely isolated MO_6_ octahedra to minimize electrostatic energy. However, the Sb_2_O_11_ dimers present in Li_4_(CuSb)O_5.5_ indicate that cationic repulsion comes only second to spreading oxygen vacancies in the structure. The SbO_6_ octahedra show little deviation from a perfectly regular octahedral environment except for a slight displacement of Sb^5+^ cations away from each other (*d*(Sb8–O6) = 2.0307(8) Å > *d*(Sb8–O5) = 1.991(5) Å). This displacement is likely the result of the minimization of electrostatic energy between the two highly charged Sb^5+^ cations. It also helps to mitigate the deviation from an ideal bond valence sum (BVS) of two for the corner-sharing oxygen (O6) at the apex of the two Sb octahedra. This oxygen site is coordinated to two Sb^5+^ and four Li^+^ cations, strongly deviating from Pauling’s rule of electroneutrality. However, it conserves a reasonable BVS of 2.028(6) thanks to relatively large bond lengths with the coordinating cations (Table S1 in SI), making it the oxygen atom with the largest octahedral volume in the structure. Finally, it can be noted that Cu and Li occupy both 6 and 5-coordinate sites with some cation mixing of Cu and Li in site 9 (36.7(2)% Li, see Table 1), site 10 (24.9(2)% Cu), and site 11 (6.1(2)% Cu), suggesting that there is little energetic difference between Li and Cu occupying the different sites. Indeed, site 10 is also significantly distorted with long apical distances (2.247(6) and 2.265(6) Å) which is favorable for Jahn–Teller Cu^2+^ ions (Table S1 in SI). The energies of several phases with different Cu/Li orderings were studied via density-functional theory (DFT) calculation, confirming that phases with Cu in sites 9 and 10 are indeed very close in energy of formation (within 0.25 eV/formula unit, see Figure S2 in SI). The structure refinement also suggests about 4.1(8)% and 9.4(6)% of oxygen vacancies in sites 5 and 6, respectively. This deviation from the stoichiometric composition and imperfect chemical ordering could point to inhomogeneities in composition and local structure in the sample, which would explain the asymmetric peak shapes associated with a distribution of cell parameters in the material.

Given the similarity between the structures of Li_2_CuO_2_, Li_5_SbO_5_, and Li_4_CuSbO_5.5_, one could expect to find a group/subgroup relationship between the three structures. This is straightforward between Li_2_CuO_2_ and Li_5_SbO_5_, with the latter belonging to a direct subgroup of the former through splitting of the Li, Cu, and O positions. The group–subgroup relationships of Li_4_CuSbO_5.5_ with Li_5_SbO_5_ and Li_2_CuO_2_ is implied by the corresponding Bragg reflections that could match the structures of Li_5_SbO_5_ (*C*2/*m*) and Li_2_CuO_2_ (*Immm*), observed when preparing Li_4_CuSbO_5.5_ at 900 °C instead of 1100 °C (Figure 1c). The refined cell volumes of these phases do not match with the parent Li_5_SbO_5_ and Li_2_CuO_2_ phases (Table S2 in SI), but instead with those of Li_4_CuSbO_5.5_ prepared at 1100 °C. Here, the group–subgroup relationship does not seem to be direct and its study would require using a large supercell of Li_5_SbO_5_ or Li_2_CuO_2_ given the more complex order found in Li_4_CuSbO_5.5_. Observing higher symmetry monoclinic and orthorhombic structures at lower temperature could point to an imperfect ordering of cations in this sample. The difference in local structure between the two samples prepared at 900 and 1100 °C was studied using two local structure probe techniques, namely X-ray pair distribution function (XPDF) and extended X-ray absorption fine structure (EXAFS). Interestingly, the local atomic arrangements investigated using XPDF (Figure S3a) show almost no difference below a 20 Å radial distance for the two samples prepared at different temperatures. This suggests that Sb, the element which scatters the strongest with X-rays, is ordered at intermediate ranges in the 900 °C sample. Clear deviations beyond 20 Å are consistent with the different average long-range structures observed from the Bragg data, which could correspond to the higher symmetry structures of Li_5_SbO_5_ and Li_2_CuO_2_. The Cu K-edge EXAFS data (Figure S3b) present largely similar local environments for Cu^2+^ cations with an excellent overlap of the two sample’s signals up to *R* = 6 Å. These two local structural probes clearly show that the local chemical environments for both materials are essentially identical, indicating that the square pyramidal configuration of Cu is strongly favored, as would be expected for this chemistry. The deviation at higher R in the EXAFS data would indicate that the long-range Cu ordering is different in Li_4_CuSbO_5.5_ when prepared at 900 and 1100 °C. Altogether, these data suggest that the ordering of cations and oxygen vacancies is incomplete at 900 °C, leading to different long-range average structural models. Higher temperature helps to overcome kinetic barriers related to structural defects and to complete the long-range ordering of the structure.

### 2.3 Cu and Sb Oxidation States

**Figure 4:**
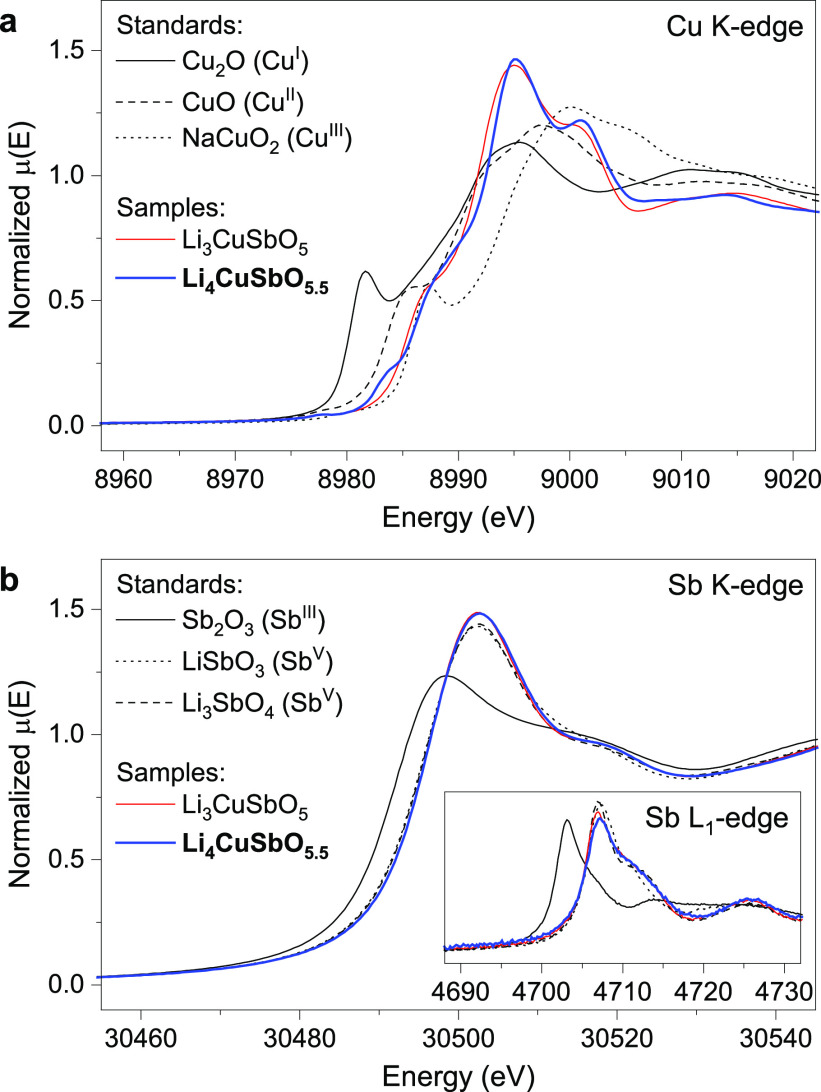
X-ray absorption near-edge spectra of Li_4_CuSbO_5.5_ and selected reference materials, measured at the Cu K-edge (a), Sb K-edge (b), and Sb L1-edge (b, inset).

Confirmation of Cu and Sb oxidation states was obtained through X-ray absorption near-edge spectroscopy (XANES) and magnetometry measurements. Measurement at the Cu K-edge (Figure 4a) was compared to reference materials with different formal oxidation states (Cu^I^_2_O, Cu^II^O, NaCu^III^O_2_) and to the reported Li_3_CuSbO_5_ phase,^11^ which contains Cu^2+^ and Sb^5+^ in comparable coordination environments. The tendency for Cu to adopt strongly distorted coordination environments clearly appears on the Cu K-edge data, with intense pre-edge features that overlap with the edge itself. Nevertheless, there is a good match between the edge position of Li_4_CuSbO_5.5_ and CuO, and even more so with Li_3_CuSbO_5_ which shows a very similar edge shape as can be expected from the comparable environments in both materials. Li_4_CuSbO_5.5_ shows an additional pre-edge feature at 8984 eV that can be interpreted as the presence of Cu^2+^ in square pyramidal environment and is not observed in Li_3_CuSbO_5_ for which all Cu occupies distorted octahedral environments. Measurements at the Sb K-edge (Figure 4b) and Sb L_1_-edge (inset), which can better resolve mixed oxidation states, unambiguously confirm a +5 valence for antimony.

**Figure 5:**
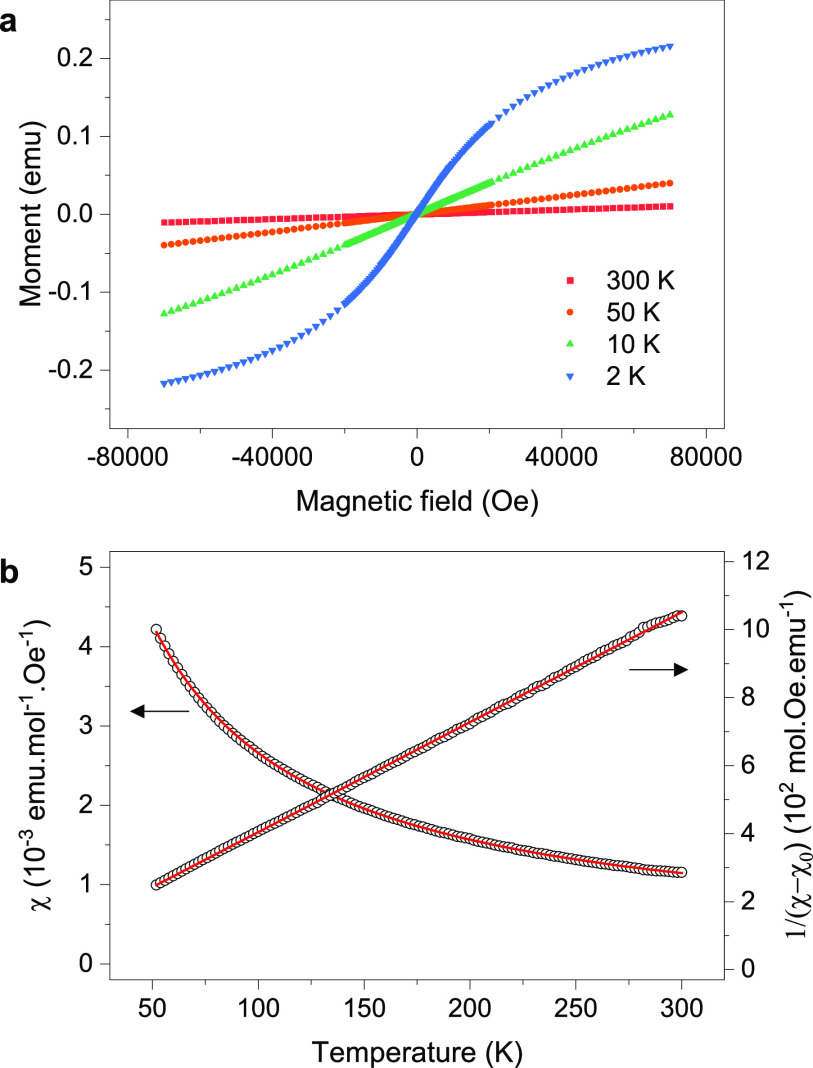
Magnetization measurements on Li_4_CuSbO_5.5_. (a) Field-dependent magnetization loops measured between 2 and 300 K. (b) Temperature-dependent magnetization curve obtained from the difference between 60 kOe and 40 kOe field-cooled measurements. Only the data above 50 K are used due to the nonlinearity of the 2 and 10 K isotherms between 40 and 60 kOe. The data are also plotted as the inverse of the magnetization after subtracting the temperature-independent paramagnetic contribution χ_0_.

Initial magnetization measurement of Li_4_CuSbO_5.5_ in an applied field of 100 Oe between 2 and 300 K shows a paramagnetic behavior which was fitted with a Curie–Weiss function modified with a temperature-independent paramagnetic contribution between 50 and 300 K. An effective magnetic moment of 1.313(3) μ_B_ per Cu atom was obtained, which is lower than the expected spin-only value for Cu^2+^ (μ_S.O._ = 1.73 μ_B_ for *S* = 1/2). Isothermal field-dependent magnetization curves measured between −70 and 70 kOe at 2, 10, 50, and 300 K (Figure 5a) show a deviation from linearity below 50 K that suggests the presence of impurities or additional magnetic interactions in the material. X-ray and neutron diffraction revealed a small amount of Li_2_CuO_2_ impurity (<1 wt %) which can affect the response of the material to the magnetic field. Even a small mole fraction of ferromagnetic impurities can affect the measurement while remaining undetectable by diffraction methods. To suppress the contribution of such impurities that saturate below 4 T, the magnetic susceptibility data measured at 60 kOe and 40 kOe were subtracted, and the data measured below 50 K were discarded (Figure 5b). This way, an effective moment of 1.571(4) μ_B_ per Cu atom is found, which is closer to the expected value for Cu^2+^.

By combining XAS and magnetization measurements, we can confirm the presence of divalent Cu in the sample, which is consistent with the detection of oxygen vacancies by neutron diffraction. The presence of defects and/or composition variations in the material may affect the magnetic susceptibility of the material. Further studies of the magnetic properties of Li_4_CuSbO_5.5_ could bring more detailed insight into these aspects.

### 2.4 Electrochemical Properties

**Figure 6:**
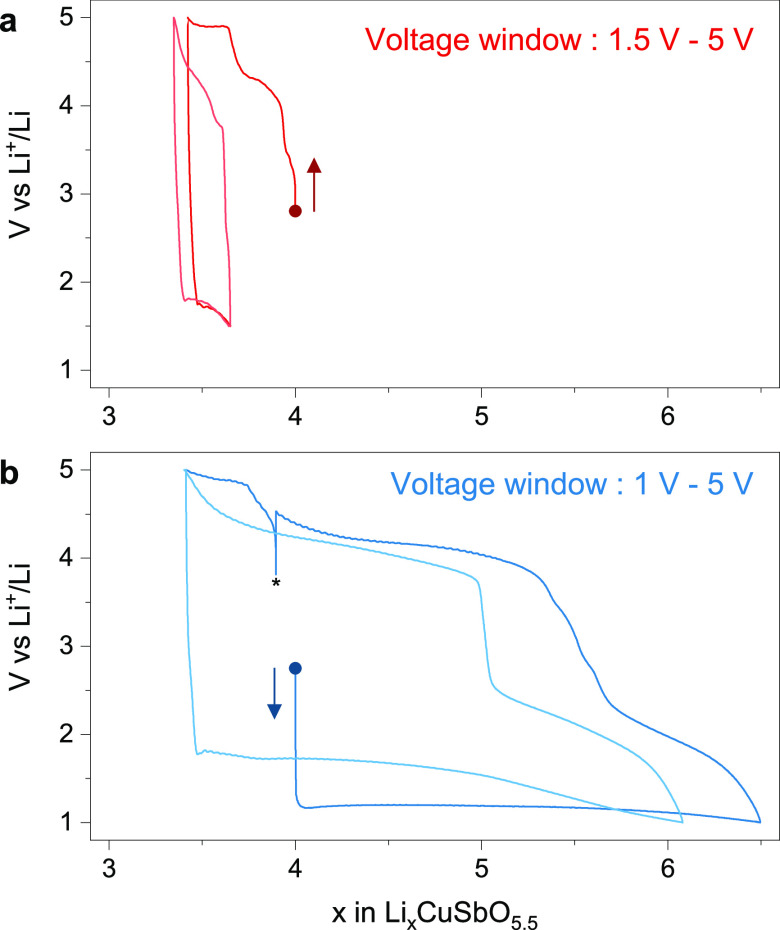
Electrochemical properties of Li_4_CuSbO_5.5_ as a cathode material in Li cells. A small capacity is obtained upon oxidation to 5 V vs Li^+^/Li (a), whereas about 2.5 Li^+^ ions can be reversibly exchanged upon reduction to 1 V vs Li^+^/Li with a large voltage hysteresis between charge and discharge (b). The first and second cycles for each cell are shown in darker and lighter colors, respectively. An asterisk marks a momentary interruption of the cycling.

The properties of Li_4_CuSbO_5.5_ as a positive electrode material in Li cells were studied at a rate of C/20 (1 Li^+^ exchanged in 20 h). Upon oxidation to 5 V vs Li^+^/Li, a charge capacity of 50 mAh/g is obtained with several features at 3.4, 4.3, and 4.9 V (Figure 6a and Figure S4a) and a reversible discharge capacity of only 20 mAh/g with a cutoff at 1.5 V vs Li^+^/Li. The theoretical capacity for the extraction of one Li^+^ ion corresponds to 90 mAh/g, suggesting that about 0.55 Li^+^ ions are extracted from the material and 0.22 are reinserted. Further cycling in this voltage range improves only slightly the capacity up to 35 mAh/g reversible capacity after 15 cycles (Figure S4b). The high-voltage plateau at 4.9 V vs Li^+^/Li is only observed on the first cycle and is reminiscent of the activation plateau of Li-rich layered oxides corresponding to irreversible oxygen oxidation. In addition to the presence of native oxygen vacancies in the pristine structure, it is possible that additional oxygen is released upon high-voltage electrochemical oxidation instead of, or concomitantly, with the oxidation of Cu^2+^ to Cu^3+^. In any case, this situation is not favorable for reversible insertion reactions.

Remarkably, when the material is discharged to 1 V vs Li^+^/Li, a large capacity reaching 222 mAh/g is obtained with a long plateau at 1.2 V (Figure 6b and Figure S4d). The next charge and discharge give a capacity of 237 and 226 mAh/g, respectively, with, however, a large voltage hysteresis (3.5 V) between discharge and charge. From the second cycle, the low voltage plateau on reduction is now found at 1.75 V instead of 1.2 V vs Li^+^/Li. A reversible capacity between 220 and 270 mAh/g was maintained for 20 cycles before observing a rapid capacity drop (Figure S4e).

To shed further light on the structural evolution of the material upon (de)lithiation, XRD data were obtained *in situ* while first charging the material to 5 V, then discharging to 1 V and charging again to 5 V (Figure S5). The capacities obtained for each step are lower than those measured in coin cells (46, 171, and 148 mAh/g, respectively), which can be explained by the larger amount of material used and higher polarization in the *in situ* cell, but they are representative enough to observe the structural evolution of the material. Very little change is observed upon the initial charge to 5 V, which is not unexpected given the small amount of Li (∼0.51) removed from the structure. Deinsertion of lithium, which is a weak X-ray scatterer, does not make an observable difference to the intensity of Bragg peaks using a lab X-ray source. However, we should be able to measure changes in lattice parameters or the creation of additional oxygen vacancies. This is not the case here. The following discharge to 1 V is marked by a strong reduction in peak intensity of all Bragg reflections corresponding to Li_4_CuSbO_5.5_. No new peaks were observed to replace them; however, a general increase of the background contribution can be noted, which suggests the formation of an amorphous phase rather than a new crystalline phase. This transition is incomplete, with some peaks from the pristine phase still observed at the end of discharge. This can be understood from the lower capacity obtained in the in situ cell (171 mAh/g) compared to the coin cell (222 mAh/g) for which the lower polarization lets the reaction proceed to completion. The second charge step shows only weak variations in the remaining peak intensity and positions as well as the background intensity. The amorphization reaction is irreversible, and the structure of Li_4_CuSbO_5.5_ is not recovered upon oxidation. The large capacity obtained is therefore likely to come from a conversion reaction that eventually leads to the cycling of an amorphous composite, thus explaining the large voltage hysteresis between discharge and charge.

## 3 Discussion

Li_4_CuSbO_5.5_ was prepared to explore the possible incorporation of oxygen vacancies in a rocksalt oxide. Before discussing this result, it is interesting to comment on the electrochemical properties of this material in light of other published work. First, we attributed the large reversible capacity on reduction to a conversion reaction that leads to an amorphous phase. It should be noted that the capacity of this reduction process (222 mAh/g) corresponds approximately to the reaction of 2.5 Li^+^ ions with Li_4_CuSbO_5.5_. This is slightly more than the capacity expected for the full reduction of Cu^2+^ to Cu^0^ (180 mAh/g), but consistent with such a reaction. Several reports have mentioned the possibility for Li^+^ to displace copper from a structure and form Cu nanoparticles.^15−18^ In a related example, Larcher et al. reported that the trirutile CuSb_2_O_6_ structure forms an amorphous Li_2_Sb_2_O_6_ phase and Cu nanoparticles upon electrochemical reduction vs metallic Li.^15^ It is possible that Li_4_CuSbO_5.5_ follows a similar reaction pathway:
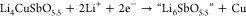
1The composition “Li_6_SbO_5.5_” does not correspond to a reported crystalline structure, and most likely forms Li_5_SbO_5_, Li_7_SbO_6_, and/or Li_2_O instead, although we cannot exclude an unknown composition for an amorphous phase. This reaction pathway could explain the short cycle life of the cell, as Li-rich pentavalent Sb phases may form an electronically insulating matrix that eventually prevents copper nanoparticles from reacting reversibly.

Second, the capacity obtained upon oxidation between 4 and 5 V vs Li^+^/Li may be explained by the Cu^2+^/Cu^3+^ redox couple or oxidation of oxygen. Multiple advanced characterization techniques would be required to fully pin down the charge compensation mechanism in this case, while maintaining a large degree of uncertainty given the small capacity associated with this process. Instead, we can probe the possibility for oxygen evolution by evaluating the Gibbs energy of eq 2, using first principle methods, with the known experimental structures for Li_4_CuSbO_5.5_, Li_3_CuSbO_5_,^11^ and Li_2_O:
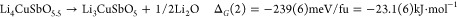
2This value can be combined with the Gibbs energy of formation of Li_2_O at 298 K (Δ_*G*_ ≈ −610 kJ·mol^–1^)^19^ to obtain the energy of eq 3, which corresponds to the deintercalation of 1 mole of lithium from Li_4_CuSbO_5.5_, oxygen release, and plating of one mole of lithium at the anode:
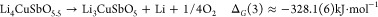
3We can therefore obtain an estimate of the average cell voltage for eq 3:
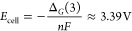
This result shows that oxygen evolution is not limited by thermodynamics, as the phase Li_3_CuSbO_5_ is known to exist, and we pushed the cell voltage well beyond 3.39 V. However, no signs of Li_3_CuSbO_5_ were observed by *in situ* XRD, which suggests that this reaction may be kinetically limited. Indeed, this reaction requires good oxygen diffusion and a large reorganization of cation and anion lattices, moving from the rocksalt-derived structure of Li_4_CuSbO_5.5_ with oxygen vacancies (density of 4.5 g·cm^–3^) to the close-packed rocksalt structure of Li_3_CuSbO_5_ (density of 4.9 g·cm^–3^).

**Figure 7:**
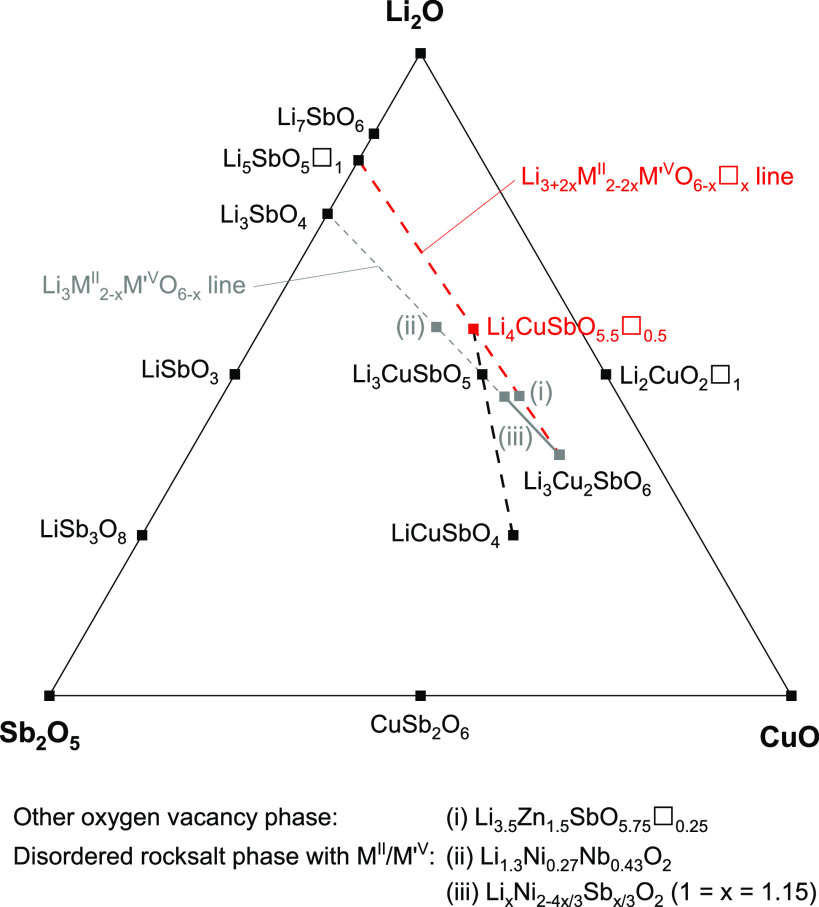
Li_2_O-CuO-Sb_2_O_5_ phase diagram. Li_4_CuSbO_5.5_ is indicated by a red marker and known phases by black markers. The black dashed line highlights the relationship between Li_4_CuSbO_5.5_, Li_3_CuSbO_5_, and LiCuSbO_4_, the red dashed line corresponds to the compositions for which the formation of oxygen vacancies is expected, including the Li_3.5_Zn_1.5_SbO_5.75_ (i) composition,^22^ and the gray dashed line corresponds to the ideal rocksalt stoichiometries with the same number of cations and anions. Representative examples of disordered rocksalt structures with divalent M^II^ and pentavalent M′^V^ cations are also indicated (ii and iii from refs (23 and 24)), showing compositions very close to the region of oxygen vacancy formation.

It is interesting to note that another phase is compositionally related to the two discussed above: the quantum-spin liquid LiCuSbO_4_,^20^ which can be obtained by removing another equivalent of Li_2_O from Li_3_CuSbO_5_ (see Figure 7). This is an example of phase dimensional reduction^21^ for which the connectivity of SbO_6_ octahedra decreases with increasing content of Li_2_O in the structure, moving from a bidimensional framework of SbO_6_ octahedra connected by edges and corners in LiCuSbO_4_ to isolated Sb_2_O_10_ dimers sharing an edge in Li_3_CuSbO_5_ and isolated Sb_2_O_11_ dimers sharing a corner only in Li_4_CuSbO_5.5_ (Figure S6 in SI). Beyond their study of intercalation materials, these compounds could present interesting magnetic properties controlled by the progressive dilution and different orderings of the *S* = 1/2 Cu^2+^ ions.

**Figure 8:**
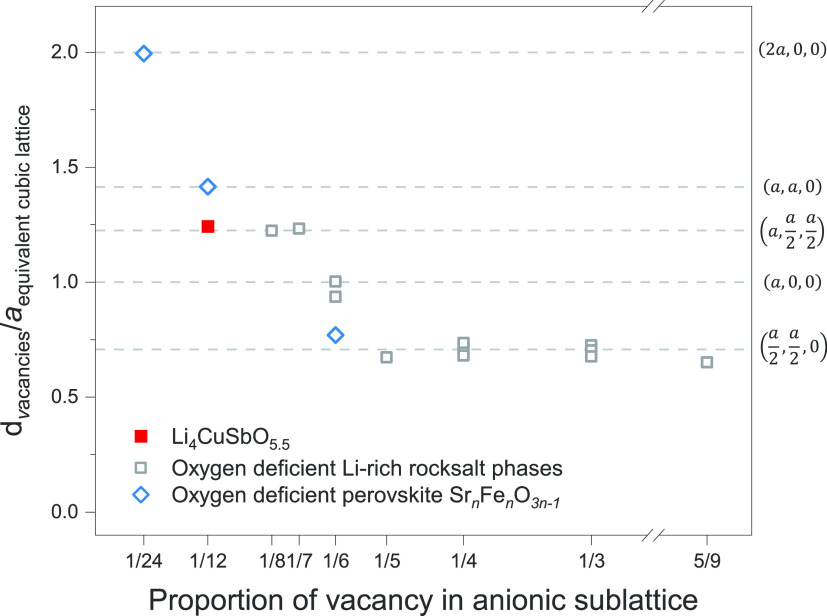
Shortest distances between vacancies in various structures with ordered oxygen sublattices. The distance is normalized by the average lattice parameter of the equivalent cubic NaCl or perovskite cells. Dashed lines indicate specific anion–anion distances in the equivalent cubic structures, with the corresponding vectors indicated on the right-hand side of the graph. The detailed values for each structure are presented in Table S3 in SI.

Focusing now on the existence of Li-rich rocksalt oxides with ordered oxygen vacancies, several materials have been reported in the literature, although not always identified as rocksalt-derived structures. Identifying such structures from an electronic database remains challenging given that we are searching for a “missing” structural feature (e.g., vacancy); therefore, some structures may have been unintentionally excluded from our search. Examples of materials with only one cation in addition to lithium and ordered oxygen vacancies are Li_2_CuO_2_, Li_2_PdO_2_, LiCu_2_O_2_, Li_3_CuO_3_, Li_3_AuO_3_, LiCu_3_O_3_, Li_3_Cu_2_O_4_, Li_5_AuO_4_, Li_5_SbO_5_, Li_5_BiO_5_, Li_6_TeO_6_, Li_6_Zr_2_O_7_, and Li_4_Cu_4_O_4_ (Table S3 in SI).^25−35^ Li_2_NiO_2_, which is structurally related to Li_2_CuO_2_, could also enter this list but is more often described as a layered rocksalt containing excess Li in tetrahedral sites.^36^ The ordering of oxygen vacancies in these structures directly depends on the proportion of unoccupied sites in the oxygen sublattice and, most of the time, allows to maximize the distance between vacancies. Examples of vacancy ordering patterns for some of these structures are shown in Figure S7 in SI. Most structures can be described by a single hexagonal anion sublattice plane stacked with a given periodicity along the [111] direction of the corresponding cubic rocksalt cell. However, Li_4_CuSbO_5.5_ requires two different alternating sublattice planes, one with a close-packed array of oxygen atoms and another with only 5 out of 6 oxygen sites occupied. Among the structures cited above, only Li_6_Zr_2_O_7_ also requires two different sublattices planes.^35^ The smallest distance between two vacancies, normalized by the average rocksalt *a* lattice parameter, is presented in Figure 8 as a function of the proportion of vacancy in the anionic sublattice. For structures with a concentration of oxygen vacancies larger than 1/6, the shortest distance between two vacancies corresponds to the shortest distance between two anionic sites in a rocksalt lattice (, corresponding to the vector  in the equivalent cubic rocksalt lattice). For structures with a concentration of oxygen vacancies smaller than 1/6, the shortest distance between two vacancies is  (corresponding to the vector  in the equivalent cubic rocksalt lattice). For a proportion of oxygen vacancy of exactly 1/6, an intermediate situation is observed: The distance between oxygen vacancies in Li_5_SbO_5_ and Li_5_BiO_5_ corresponds to the *a* lattice parameter in the cubic parent. Li_4_CuSbO_5.5_, with 1 out of 12 oxygen sites in the unit cell being vacant, is the rocksalt structure with the smallest amount of ordered oxygen vacancy we were able to identify. It is noteworthy that Sr_8_Fe_8_O_23_, derived from the perovskite structure,^37^ maintains ordered oxygen vacancies with as little as 1 in 24 sites unoccupied. In that case, vacancies are separated by a distance of 2 × *a*, corresponding to the (2*a*, 0, 0) vector in the equivalent perovskite lattice. Sr_4_Fe_4_O_11_ and Sr_2_Fe_2_O_5_, the *n* = 2 and 4 members of the oxygen-deficient Sr_*n*_Fe_*n*_O_3*n*–1_ series,^37^ follow a similar trend and can be presented in Figure 8 for comparison, as the lattice parameter *a* and vectors discussed above are also relevant in the cubic perovskite lattice. Given this comparison, it cannot be excluded that Li-rich rocksalt oxides with more complex compositions could be prepared with a lower concentration of ordered oxygen vacancies than in Li_4_CuSbO_5.5_. In that regard, it should be noted that Greaves and Katib^22^ reported the preparation of Li_3.5_Zn_1.5_SbO_5.75_□_0.25_ and Li_3.5_Zn_1.5_BiO_5.75_□_0.25_, that is, 1 in 24 oxygen sites unoccupied, with, however, oxygen vacancies statistically distributed over all oxygen positions. This result shows that (i) other divalent M^II^/pentavalent M′^V^ cation couples may be used to prepare Li-rich rocksalt oxides with oxygen vacancies and (ii) the amount of oxygen vacancies in Li_3+2*x*_M^II^_2–2*x*_M′^V^O_6–*x*_□_*x*_ may vary between Li_3_M^II^_2_M′^V^O_6_ (*x* = 0) and Li_5_M′^V^O_5_□_1_ (*x* = 1) (Figure 7). This large chemical versatility is further supported by an early report from Brixner^38^ of oxygen-deficient cubic rocksalt phases based on Nb/Ta pentavalent cations and Mn/Fe/Co/Ni/Cu/Zn divalent cations. Altogether, it appears that the presence of oxygen vacancies in Li-rich rocksalt oxides is not limited to oxygen release induced by electrochemical delithiation but may already exist in the pristine state of materials after their synthesis to balance ionic charges and achieve charge neutrality. Synthesis conditions (temperature, partial pressure of oxygen) that favor the formation of trivalent cations tend to give the well-known layered honeycomb Li_5_ReO_6_-type structure, as is the case for Cr, Al, and Ga compounds.^39^ However, by using reducing conditions to stabilize divalent cations, we can expect the formation of oxygen vacancies with other transition metals (Mn, Fe, Co, Ni) beyond Cu and Zn-based compounds for which normal atmospheric conditions are sufficient. Two-step synthesis procedures, starting with the reaction of precursors at high temperature, followed by a second annealing step under oxidizing or reducing conditions, have been successful in the preparation of double perovskites with controlled oxygen vacancy content (e.g., Sr_2_CoSbO_6–*x*,_Ca_2_MnNbO_6–*x*_).^40−42^ Transposing this approach to the preparation of Li-rich rocksalt oxides could lead to a better understanding of defect formation in this structural family. Reciprocally, the discovery of Li-rich rocksalt oxides Li_4_MM′O_6–*x*_ can be used to prepare functional double perovskite A_2_MM′O_6–*x*_ (A = alkaline-earth) by simple metathesis solid-state routes using ACl_2_ salts.^43,44^ This approach enables the formation of double perovskite with new cation ordering patterns at reduced temperatures (700–900 °C) compared to conventional synthesis routes (>1000 °C).

Formation of oxygen vacancies is not the only mechanism to balance ionic charges in Li-based rocksalt structures. Another known mechanism is the formation of cation vacancies (lithium and/or transition metals) as studied for materials such as Li(Ni_1/6_□_1/6_Mn_2/3_)O_2_^45^ and phases derived from the Li_4_FeSbO_6_ composition.^46^ These examples pointed out that cation vacancies in a rocksalt structure are found when an overstoichiometry of anions compared to metal atoms is required to ensure electroneutrality. This is more likely to be achieved in relatively oxidizing conditions, in opposition with the formation of structures with oxygen vacancies.

Overall, a better understanding of the formation of oxygen vacancies at the synthesis step in Li-rich rocksalt oxides could greatly benefit the search for advanced cathode materials for Li-ion batteries. This is particularly true concerning the recent focus on disordered rocksalt oxides, made of divalent cations and d^0^ cations such as Nb^5+^, Sb^5+^, Ta^5+^ to increase the Li content.^23,24^ The cationic disorder in those compounds is statistically expected to yield oxygen sites with a higher redox activity, but it could also result in sites containing oxygen vacancies given that their average composition is close to the oxygen-vacancy structure domain (Figure 7). Integrating the possibility for the presence of oxygen vacancies in theoretical models and structural analysis for this family of material may help to shed new light on their complex charge compensation mechanisms.

## 4 Conclusion

We report the synthesis and crystal structure of a new Li_4_CuSbO_5.5_□_0.5_ phase containing ordered oxygen vacancies, which are confirmed by the combined Rietveld refinement of synchrotron and neutron powder diffraction data and indirectly from spectroscopic evidence for divalent copper. Only one in 12 oxygen sites is unoccupied in the lattice, which represents the lowest concentration of ordered oxygen vacancies reported in a Li-rich rocksalt oxide. The cation and oxygen sites order to maximize the distance between vacancies, requiring two inequivalent hexagonal sublattice planes to fully describe the vacancy order. Cationic repulsion between highly charged cations comes only second to the ordering of oxygen vacancies. The electrochemical properties of this material as a cathode material in a Li cell present interesting features, despite performances which are not competitive with other compositions. More importantly, this work offers a new insight into the crystal chemistry of Li-rich rocksalt oxides, namely the possible presence of oxygen vacancies in as-prepared materials, which can have a strong effect on the understanding of the charge compensation mechanism in high-energy density cathode materials.

## 5 Methods

### 5.1 Synthesis

Li_4_CuSbO_5.5_ is prepared from Li_2_CO_3_ (Sigma-Aldrich, 99.99% trace metals basis), CuO (Sigma-Aldrich, 99.99% trace metals basis), and Sb_2_O_3_ (Sigma-Aldrich, 99.99% trace metals basis). All precursors are kept in a drying oven at 200 °C before use. The precursors in the proportions Li:Cu:Sb = 4.4:1:1 are either hand ground or ball-milled using a planetary mill with the same outcome. They are then fired in air at 1100 °C for 12 h (1 °C/min heating ramp, cooling inside the furnace turned off). The excess of Li_2_CO_3_ used plays a role in stabilizing Li_4_CuSbO_5.5_ as longer heating at 1100 °C will result in Li_2_O loss from the compound to form Li_3_CuSbO_5_ according to the reaction Li_4_CuSbO_5.5_ → Li_3_CuSbO_5_ + 1/2 Li_2_O. Rapidly quenching the sample from 1100 °C between stainless steel plates or slowly cooling (0.1 °C/min) it from 850 °C to room temperature leads to small variations in cell parameters of the sample (see Table S2 in SI). The low-temperature sample was prepared with the same procedure, using a firing temperature of 900 °C instead of 1100 °C. For structural characterization by synchrotron X-ray and neutron diffraction, a sample was prepared using enriched ^7^Li_2_CO_3_ (99% ^7^Li atom, Sigma-Aldrich) to reduce the absorption of neutrons due to ^6^Li in the sample. The material was manipulated in air but kept in an Ar-filled glovebox during storage to prevent building a Li_2_CO_3_ layer at the surface with prolonged contact with ambient air.

### 5.2 Diffraction

Routine analysis of phase purity and lattice parameters were performed on a Panalytical diffractometer with a monochromatic Co source (Kα_1_, λ = 1.78901 Å) in Bragg–Brentano geometry with sample rotation. SXRD was performed at the I11 beamline at Diamond Light Source (Oxfordshire, UK), with an incident wavelength of 0.82468(1) Å using five multianalyzer crystal detectors. The samples were sealed in Ø = 0.3 mm glass capillaries and spun during measurement. Time-of-flight (ToF) neutron powder diffraction data were collected on the HRPD instrument at ISIS neutron source (Oxfordshire, UK). Samples were sealed in Ø = 6 mm vanadium cylindrical cans in an argon-filled glovebox. Indexing was performed using the DICVOL method as implemented in the Fullprof suite.^14^ A simulated annealing method was used to obtain initial atomic positions with the FOX program.^13^ The structural model was completed and refined by the Rietveld method^47,48^ and using Fourier difference maps as implemented in the Fullprof suite.^14^ In situ XRD was performed using an electrochemical cell equipped with a Be window (250 μm thick) and an Al current collector (3 μm thick) on a Rigaku SmartLab diffractometer with a 9 kW rotating anode providing a parallel beam of Mo Kα_1_ radiation (λ_Kα1_ = 0.709032 Å). X-ray total scattering measurements were done at the I15-1 (XPDF) beamline at Diamond Light Source (Oxfordshire, UK). Samples were loaded into quartz capillaries with a 1 mm inside diameter and measured while spinning. Data were collected using a PerkinElmer XRD1611 CP3 area detector with an active area of 409.6 × 409.6 mm^2^ with a *Q* range of 36 Å^–1^. Pair distribution functions were calculated using GudrunX using the appropriate composition, background data, instrument corrections, and a *Q* max of 30 Å^–1^.

### 5.3 Electrochemical Characterization

Electrochemical characterization was performed in two-electrode Swagelok cells and 2025-type coin cells. The positive electrode consisted of a laminated mixture of active material (Li_4_CuSbO_5.5_), conductive carbon (C65 from Timcal), and binder (polytetrafluoroethylene, PTFE dried from a 60% aqueous suspension from Sigma-Aldrich) in proportions 85:10:5 in weight. For ex situ characterization by XRD, the active material was simply mixed with 10 wt % C65 conductive carbon and used as a powder. Active material loadings were typically between 5 and 10 mg. Metallic Li was used as an anode, LP30 (1 M LiPF_6_ in EC:DMC 1:1) was used for the electrolyte, and Whatman GF/D borosilicate glass fiber membranes dried under vacuum at 300 °C for 24 h as the separator. All parts were assembled in an Ar-filled glovebox. Galvanostatic cycling was performed at a C/20 rate (defined as 1 Li^+^ exchanged in 20 h, considering the chemical formula Li_4_CuSbO_5.5_) between 1 and 5 V vs Li^+^/Li. After cycling, samples for ex situ characterization were recovered inside the glovebox, washed three times in anhydrous DMC, and dried under vacuum.

### 5.4 X-ray Absorption Spectroscopy

Pellets of sample diluted with cellulose were prepared with an optimized density for X-ray absorption measurements at the Cu K-edge, Sb K-edge, and Sb L1-edge. X-ray absorption spectra were measured at the B18 beamline at Diamond Light Source (Oxfordshire, UK) in transmission mode. The spectra were calibrated by fixing at 8979/30491/4966 eV, the maximum of the derivative of Cu/Sb/Ti metal foil references placed after the samples for the corresponding edges, respectively, and normalized with the Athena software.^49^ Fourier transform of the EXAFS Cu K-edge data was done using a sine window function from 3.8 to 12.7 Å^–1^ (*k*-range).

### 5.5 Magnetization Measurement

About 30–40 mg of freshly prepared sample in the form of a powder was sealed in a high purity quartz tube (Suprasil Medium Wall EPR Tubes, Goss Scientific). Magnetic measurements were carried out using a commercial superconducting quantum interference device magnetometer MPMS3 (Quantum Design, USA). The contribution of the quartz tube to the magnetization was confirmed to be negligible prior to measuring the sample. Zero-field cooled and field cooled measurements were first performed at 100 Oe from 2 to 300 K, followed by field-cooled measurements at 40 kOe and 60 kOe. Finally, magnetic field-dependent magnetization *M*(H) loops were measured between −70 and 70 kOe at 2, 10, 50, and 300 K.

### 5.6 Computational Methods

All calculations were conducted with DFT as implemented in VASP-5.4.4^50^ with PAW pseudopotentials.^51^ The atomic and vacancy positions in the structures with mixed site occupancies, Li_4_CuSbO_5.5_ and Li_3_CuSbO_5_ were determined by, first, optimizing the geometries of possible atomic configurations in 2 × 2 × 1 supercell structures (obtained with Supercell program^52^ from experimentally obtained atomic positions in a unit cell), the interatomic forces are reduced below 10^–3^ eV/Å, and then comparing their total energies. The lowest energies were used in eqs 2 and 3. Calculations were performed with a 700 eV kinetic energy cutoff for plane waves, 5 × 5 × 5 *k*-points sampling, and LDSA^53^ approach to account for strongly correlated 3d electrons in Cu, with Hubbard *U* = 9.79 eV and *J* = 2.5.^54^

## Supporting Information Available

Details on the structure of Li_4_CuSbO_5.5_ obtained by Rietveld refinement and used for DFT calculation. PDF and EXAFS data on low- and high-temperature samples of Li_4_CuSbO_5.5_. Capacity retention upon long-term electrochemical cycling. In situ XRD data during cycling. Comparison of structures with ordered oxygen vacancies (PDF)

## Accession Codes

CCDC 2108517 contains the supplementary crystallographic data for this paper. These data can be obtained free of charge via www.ccdc.cam.ac.uk/data_request/cif, or by emailing data_request@ccdc.cam.ac.uk, or by contacting The Cambridge Crystallographic Data Centre, 12 Union Road, Cambridge CB2 1EZ, UK; fax: +44 1223 336033.
