# Supplementary material for: Ordered Oxygen Vacancies in the Lithium-Rich Oxide Li4CuSbO5.5, a Triclinic Structure Type Derived from the Cubic Rocksalt Structure
Source: Inorg Chem. 2021 Dec 6;60(24):19022–34. doi: 10.1021/acs.inorgchem.1c02882 (PMC8693191; doi:10.1021/acs.inorgchem.1c02882)
Supplement: Supplementary file 1 — ic1c02882_si_001.pdf [file ic1c02882_si_001.pdf]

Supplementary Information

Ordered oxygen vacancies in the lithium-rich oxide $Li_4CuSbO_{5.5}$, a triclinic structure type derived from the cubic rocksalt structure.

Arnaud J. Perez[1], Andrij Vasylenko[1], T. Wesley Surta[1], Hongjun Niu[1], Luke M. Daniels[1], Laurence J. Hardwick[1,2], Matthew S. Dyer[1], John B. Claridge[1] and Matthew J. Rosseinsky[1,*]

[1]Department of Chemistry, University of Liverpool, Crown Street, Liverpool, L69 7ZD, United Kingdom

[2]Stephenson Institute for Renewable Energy, University of Liverpool, Chadwick Building, Peach Street, Liverpool, L69 7ZF, United Kingdom

[*] Corresponding author: rossein@liverpool.ac.uk

List of tables

Table S1: Interatomic distances and bond valence sums in $Li_4CuSbO_{5.5}$. 3

Table S2: Comparison of cell parameters. 4

Table S3: Example of oxides with ordered oxygen vacancies derived from the rocksalt and perovskite cubic structures. 5

List of figures

Figure S1: Structure obtained from a Rietveld refinement of synchrotron and neutron diffraction data for $Li_4CuSbO_{5.5}$...6

Figure S2: Optimized DFT structures used to explore different cation orderings in the rocksalt structure for the formula $Li_4CuSbO_{5.5}$. 7

Figure S3: X-ray Pair Distribution Function ($D(r)$) (a) and Cu K-edge EXAFS (b) data for $Li_4CuSbO_{5.5}$ 8

Figure S4: Electrochemical data on $Li_4CuSbO_{5.5}$ 9

Figure S5: *In situ* X-ray diffraction experiment on $Li_4CuSbO_{5.5}$. 10

Figure S6: Variation in octahedra connectivity of related phases.............................11

Figure S7: Ordering of oxygen vacancies in Li-rich rocksalt oxides 12

Table S1: Interatomic distances and bond valence sums in $Li_4CuSbO_{5.5}$.

Atom 1	Atom 2	Distance (Å)	BVS	Atom 1	Atom 2	Distance (Å)	BVS
O1	Sb8	1.984(6)	2.1(2)	Sb8	O1	1.984(6)	5.16(3)
	Cu9/Li9	2.012(7)			O2	1.984(6)	
	Li12	1.886(18)			O3	1.986(5)	
	Li12	2.044(16)			O4	1.964(6)	
	Cu11/Li11	2.183(13)			O5	1.991(5)	
	Li13	2.173(15)			O6	2.0307(8)	
O2	Sb8	1.984(6)	2.1(2)	Cu9/Li9	O1	2.012(7)	2.165(13)/ 1.217(7)
	Cu9/Li9	1.975(6)			O2	1.975(6)	
	Cu10/Li10	2.249(6)			O3	2.045(7)	
	Li12	1.95(2)			O4	1.992(6)	
	Cu11/Li11	2.011(10)			O5	2.288(5)	
	Cu11/Li11	2.123(11)					
O3	Sb8	1.986(5)	1.88(17)	Cu10/Li10	O2	2.247(6)	1.644(12)/ 0.925(7)
	Cu9/Li9	2.045(7)			O3	2.140(8)	
	Cu10/Li10	2.140(8)			O3	2.265(6)	
	Cu10/Li10	2.265(6)			O4	2.138(7)	
	Li12	2.062(15)			O5	2.065(7)	
	Li13	2.165(18)			O5	2.097(8)	
O4	Sb8	1.964(6)	2.03(19)	Cu11/Li11	O1	2.183(13)	1.89(2)/ 1.063(12)
	Cu9/Li9	1.992(6)			O2	2.009(10)	
	Cu10/Li10	2.139(7)			O2	2.124(11)	
	Cu11/Li11	1.974(10)			O4	1.975(10)	
	Li13	2.14(2)			O6	2.173(11)	
	Li13	2.285(15)					
O5	Sb8	1.991(5)	1.81(16)	Li12	O1	1.887(18)	1.13(2)
	Cu9/Li9	2.288(5)			O1	2.046(16)	
	Cu10/Li10	2.064(7)			O2	1.95(2)	
	Cu10/Li10	2.096(8)			O3	2.061(15)	
	Li13	2.097(17)			O6	2.439(16)	
	Li13	2.206(19)					
O6			2.028(6)	Li13	O1	2.171(15)	0.873(17)
	Sb8 (x2)	2.0307(8)			O3	2.165(18)	
	Li12 (x2)	2.442(16)			O4	2.14(2)	
	Cu11/Li11 (x2)	2.173(11)			O4	2.286(15)	
					O5	2.097(17)	
					O5	2.206(19)	
O7 - vacancy	Cu9/Li9 (x2)	2.097(3)	0.963(7)[a]				
	Cu11/Li11 (x2)	2.212(10)					
	Li12 (x2)	2.455(19)					

[a] Hypothetical value if site 1*b* is filled with an oxygen atom

Table S2: Comparison of cell parameters. The $Li_4CuSbO_{5.5}$ sample prepared at 900°C can be indexed using a three-phase model ($Immm$, $C2/m$ and $P\bar{1}$ structures) of the related Li_2CuO_2, Li_5SbO_5 and $Li_4CuSbO_{5.5}$ (1100°C) materials, with cell volumes matching that of the $Li_4CuSbO_{5.5}$ sample prepared at 1100°C. Cell parameters of samples quenched from 1100°C to room temperature and slowly cooled from 850°C to room temperature are also indicated for information.

Material	Space group	Z	*a* (Å)	*b* (Å)	c (Å)	*α (°)*	*β (°)*	*γ (°)*	Volume/Z (Å^3)
**Li_2CuO_2**	$Immm$	1	9.393	3.662	2.863	90	90	90	98.45
**Li_5SbO_5**	$C2/m$	2	9.724	3.984	5.792	90	109.04	90	106.05
**$Li_4CuSbO_{5.5}$ (900°C)**	$Immm$	1	9.028	4.200	2.908	90	90	90	110.28
	$C2/m$	2	9.497	4.203	5.817	90	108.16	90	110.31
	$P\bar{1}$	2	5.203	5.816	7.884	100.50	96.94	106.89	110.30
**$Li_4CuSbO_{5.5}$ (1100°C) - main**	$P\bar{1}$	2	5.207	5.817	7.888	100.58	96.94	106.96	110.37
**$Li_4CuSbO_{5.5}$ (1100°C) - secondary**	$P\bar{1}$	2	5.2026	5.8200	7.8800	100.469	97.015	107.025	110.21
**$Li_4CuSbO_{5.5}$ (1100°C) - quenched**	$P\bar{1}$	2	5.2048	5.8230	7.8904	100.530	96.812	107.098	110.45
**$Li_4CuSbO_{5.5}$ (1100°C) – slowly cooled**	$P\bar{1}$	2	5.2021	5.8184	7.8846	100.539	96.895	106.957	110.29

Table S3: Examples of oxides with ordered oxygen vacancies derived from the rocksalt and perovskite cubic structures. d_{vac} is the shortest distance between two vacant oxygen sites in the structure. To compare this value between materials and to relate it to vectors in the anionic sublattice, it is normalized by the lattice parameter *a* of the equivalent cubic lattice. The latter is calculated as the cube root of the volume of the equivalent cubic lattice given the lattice volume, number of atoms per formula unit and number of formula unit per unit cell Z for each compound.

**Material**	**Vacancy /f.u.**	**Proportion of vacant oxygen site**	**d_{vac} (Å)**	**$V_{equivalent\ cubic\ cell}$ (Å^3)**	**$a_{equivalent\ cubic\ cell}$ (Å)**	**$d_{vac}/a_{equivalent\ cubic\ lattice}$**	**Ref**
$(Li_4Cu_4□_1)(O_4□_5)$	5	5/9	2.672	69.028	4.102	0.651	[1]
$Li_2CuO_2□_1$	1	1/3	2.863	65.613	4.033	0.710	[2]
$Li_2PdO_2□_1$	1	1/3	2.982	69.507	4.112	0.725	[3]
$LiCu_2O_2□_1$	1	1/3	2.869	67.843	4.079	0.703	[4,5]
$Li_5AuO_4□_2$	1	1/3	2.940	82.112	4.346	0.676	[6]
$Li_3CuO_3□_1$	1	1/4	2.907	67.898	4.080	0.713	[7]
$Li_3AuO_3□_1$	1	1/4	3.092	74.210	4.202	0.736	[6]
$LiCu_3O_3□_1$	1	1/4	2.810	70.190	4.125	0.681	[4,5]
$Li_3Cu_2O_4□_1$	1	1/5	2.777	70.108	4.123	0.674	[8]
$Li_5SbO_5□_1$	1	1/6	3.984	77.157	4.257	0.936	[9]
$Li_5BiO_5□_1$	1	1/6	4.148	70.703	4.135	1.003	[9]
$Li_6TeO_6□_1$	1	1/7	5.128	72.011	4.160	1.233	[10]
$Li_6Zr_2O_7□_1$	1	1/8	5.239	78.458	4.281	1.224	[11]
**$Li_8Cu_2Sb_2O_{11}□_1$**	**1**	**1/12**	**5.207**	**73.595**	**4.191**	**1.243**	This work
$Sr_2Fe_2O_5□_1$	1	1/6	3.032	61.091	3.938	0.770	[12]
$Sr_4Fe_4O_{11}□_1$	1	1/12	5.473	57.824	3.867	1.415	[12]
$Sr_8Fe_8O_{23}□_1$	1	1/24	7.698	57.467	3.859	1.995	[12]

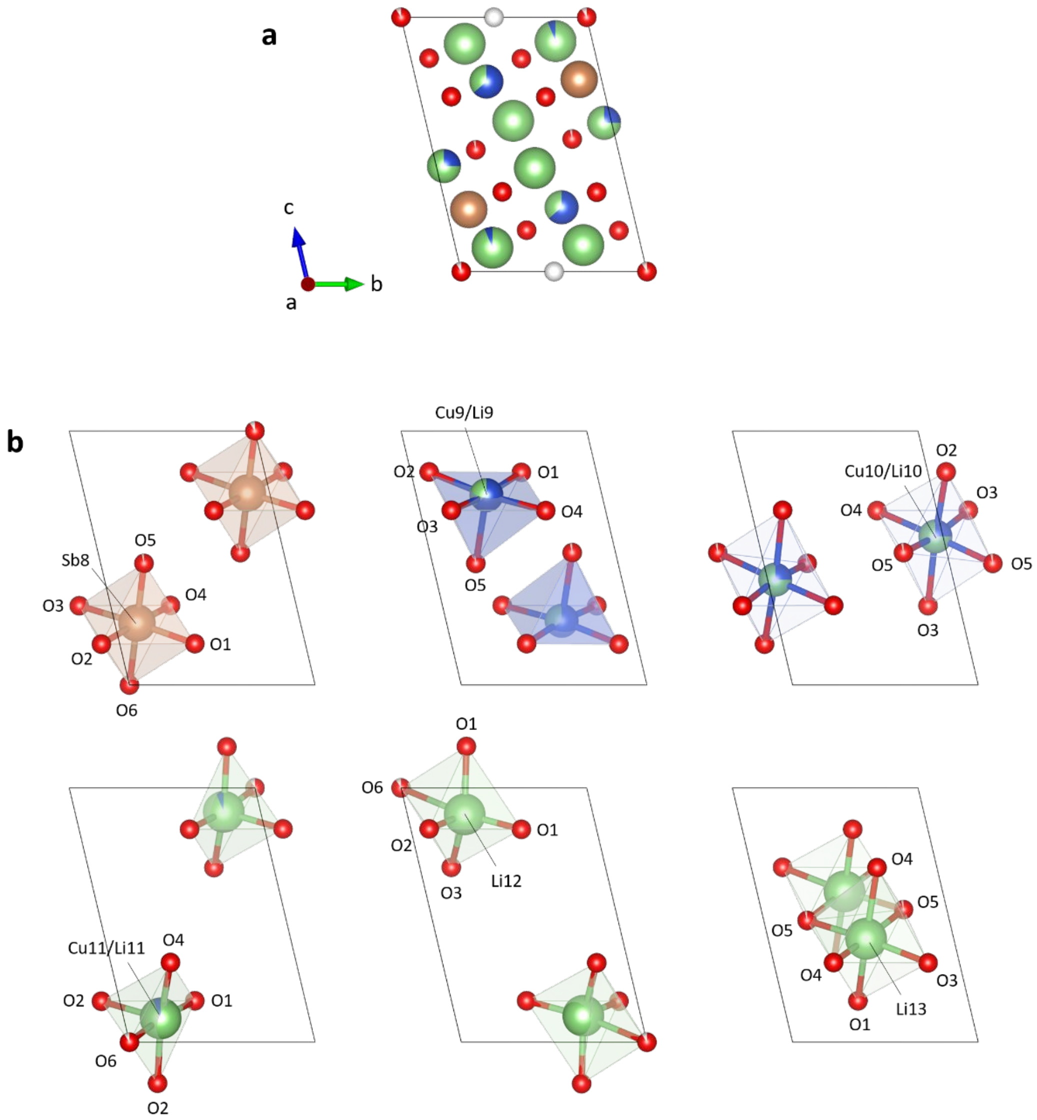

Figure S1: Structure obtained from a Rietveld refinement of synchrotron and neutron diffraction data for $Li_4CuSbO_{5.5}$. (a) Structure represented along the *a* axis. Oxygen atoms are represented in red, oxygen vacancies in white, antimony in brown, copper in blue and lithium in green. (b) Individual cationic environments in the structure. Corresponding interatomic distances are listed in Table S1.

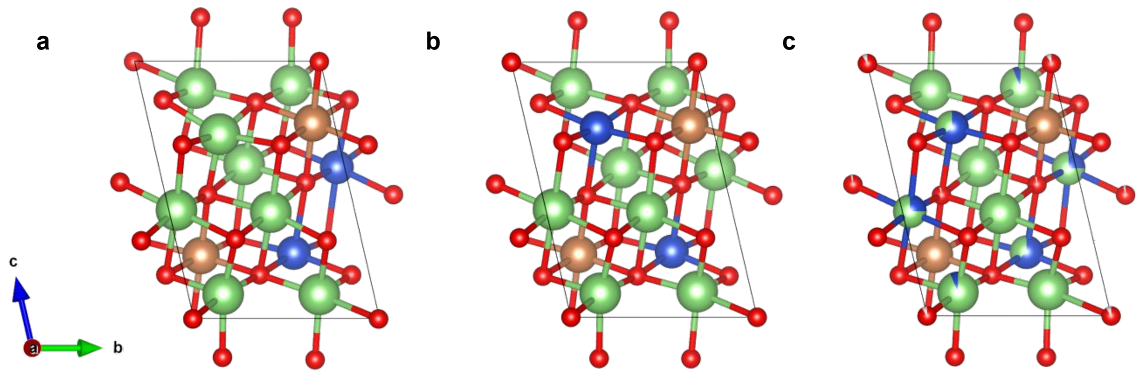

Figure S2: Optimized DFT structures used to explore different cation orderings in the rocksalt structure for the formula $Li_4CuSbO_{5.5}$. (a) Lowest energy (-60.0163 eV/formula unit) and (b) second to lowest energy (-59.7636 eV/formula unit) structures are compared to (c) the experimental structure. Antimony, copper, lithium and oxygen atoms are in brown, blue, green and red respectively.

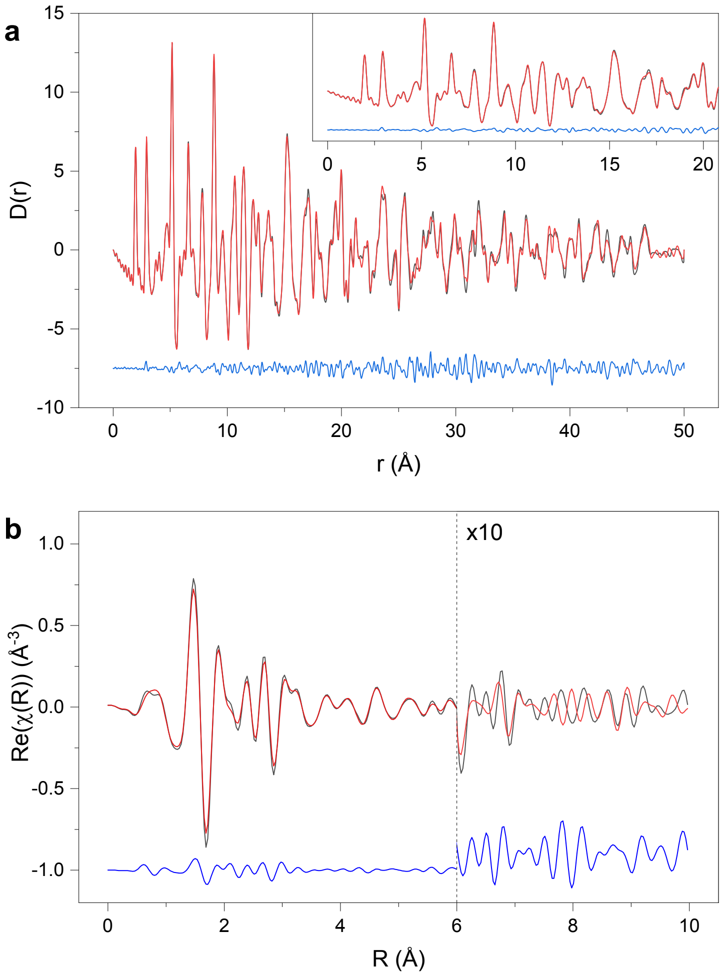

Figure S3: X-ray Pair Distribution Function (*D*(*r*)) (a) and Cu K-edge EXAFS (b) data for $Li_4CuSbO_{5.5}$ prepared at 900°C (red) and at 1100°C (black). The blue line represents the difference between the two samples. The inset in (a) highlights the excellent overlap between the two samples below the radial distance r = 20 Å whereas deviations are observed beyond that value. The Cu K-edge EXAFS (b) data also shows a good overlap below R = 6 Å and deviations at larger R values.

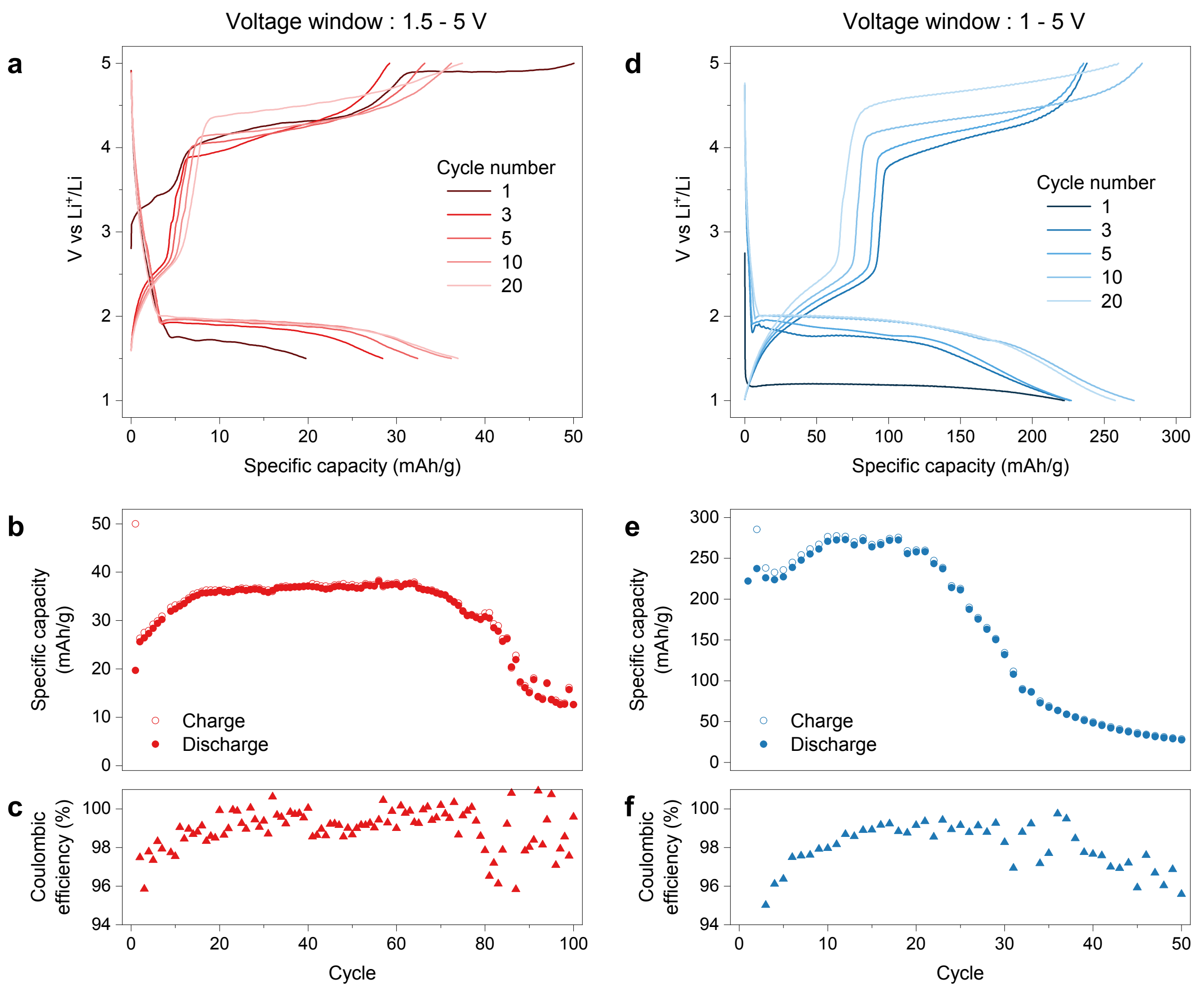

Figure S4: Electrochemical data on $Li_4CuSbO_{5.5}$ cycling between 1.5 and 5 V vs Li^+/Li, starting on charge (a, b, c) or between 1 and 5 V vs Li^+/Li, starting on discharge (d, e, f). Voltage curves of selected cycles are presented (a, d), with the evolution of the specific capacity (b, e) and coulombic efficiency (c, f) until failure of the cells.

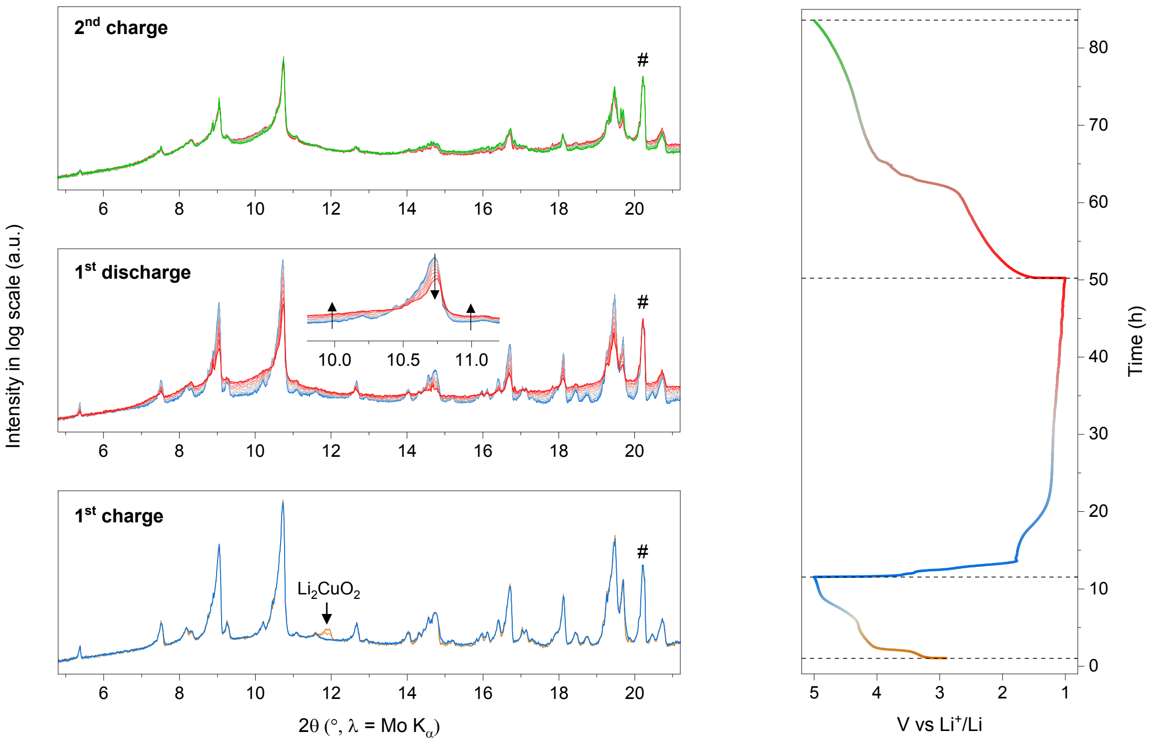

Figure S5: *In situ* X-ray diffraction experiment on $Li_4CuSbO_{5.5}$. No change is observed during the initial charge to 5 V vs Li^+/Li except for the disappearance of a peak attributed to a Li_2CuO_2 impurity. Discharge to 1 V vs Li^+/Li results in a decrease of peak intensity for all Bragg reflections belonging to $Li_4CuSbO_{5.5}$, together with an increase of the background intensity. This transition is probably incomplete due to the large loading of material used in the *in situ* cell. Finally, a second charge to 5 V vs Li^+/Li shows slight variations in intensity, but nothing that indicates a reversible process. The marked reflection at 20.2° belongs to the *in situ* cell and does not change upon cycling. The intensity of diffraction pattern is plotted in logarithmic scale to help observing the less intense peaks and change in background intensity.

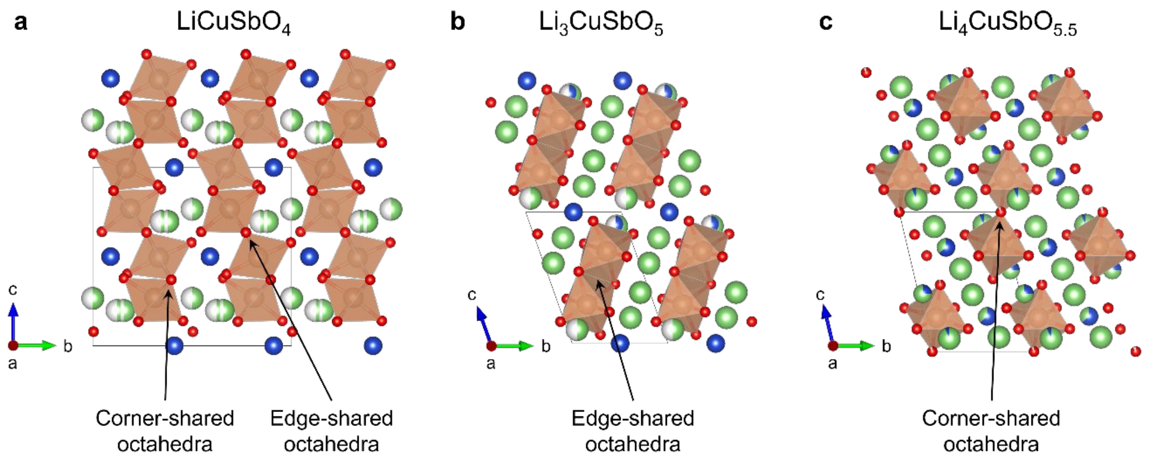

Figure S6: Variation in octahedra connectivity of related phases: (a) LiCuSbO4 presents a bidimensional framework of SbO_6 octahedra connected by edges and corners, (b) Li_3CuSbO_5 is made of isolated Sb_2O_{10} dimers composed of two edge-sharing octahedra and (c) $Li_4CuSbO_{5.5}$ is made of isolated Sb_2O_{11} dimers composed of two corner-sharing octahedra. Cu and Li are partially disordered in Li_3CuSbO_5 and $Li_4CuSbO_{5.5}$. In $LiCuSbO_4$ however, Cu is occupying a single site and the positions of Li atoms are splitted with a statistical occupation of each site.

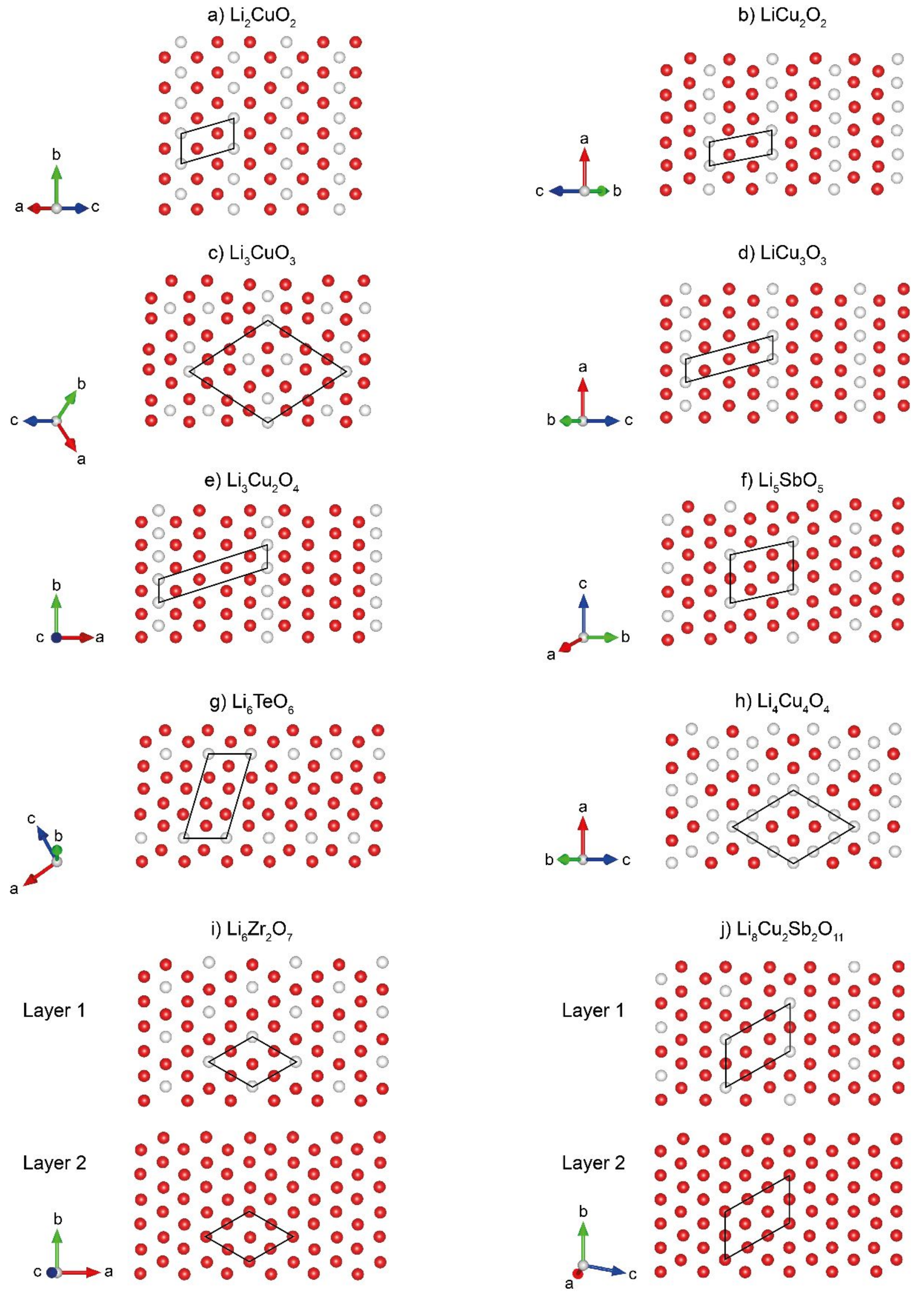

Figure S7: Ordering of oxygen vacancies in Li-rich rocksalt oxides. The anionic sublattice of the rocksalt structure can be described by a unique hexagonal close-packed layer of oxygen atoms. Depending on the proportion of oxygen vacancies in the structure, different orderings of occupied (red) and unoccupied (white) oxygen sites are observed. For Li_2CuO_2 (a), $LiCu_2O_2$ (b), Li_3CuO_3 (c), $LiCu_3O_3$ (d), $Li_3Cu_2O_4$ (e), Li_5SbO_5 (f), Li_6TeO_6 (g) and $Li_4Cu_4O_4$ (h) a unique layer is necessary to describe this ordering. However, for structure with a lower proportion of oxygen vacancies such as $Li_6Zr_2O_7$ (i) and $Li_4CuSbO_{5.5}$ (j), two different layers stacked alternatively are needed. The 2D lattices presented here are guides for the reader but do not represent the actual 3D lattice of the corresponding materials.

References

(1) Migeon, H.-N.; Zanne, M.; Gleitzer, C.; Courtois, A. Préparation et Étude de LiCuO. *Journal of Solid State Chemistry* **1976**, *16* (3–4), 325–330.

(2) Hoffmann, R.; Hoppe, R.; Schäfer, W. Neutronenbeugung an Li_2CuO_2. *Zeitschrift für anorganische und allgemeine Chemie* **1989**, *578* (1), 18–26.

(3) Wolf, R.; Hoppe, R. Notiz über Li_2PdO_2. *Zeitschrift für anorganische und allgemeine Chemie* **1986**, *536* (5), 77–80.

(4) Hibble, S. J.; Köhler, J.; Simon, A.; Paider, S. $LiCu_2O_2$ and $LiCu_3O_3$: New Mixed Valent Copper Oxides. *Journal of Solid State Chemistry* **1990**, *88* (2), 534–542.

(5) Berger, R.; Önnerud, T.; Tellgren, R. Structure Refinements of $LiCu_2O_2$ and $LiCu_3O_3$ from Neutron Powder Diffraction Data. *Journal of Alloys and Compounds* **1992**, *184* (2), 315–322.

(6) Wasel-Nielen, H.-D.; Hoppe, R. Zur Kristallstruktur von Li_3AuO_3, Li_5AuO_4, $KAuO_2$ und $RbAuO_2$. *Zeitschrift für anorganische und allgemeine Chemie* **1970**, *375* (1), 43–54.

(7) Migeon, H.-N.; Courtois, A.; Zanne, M.; Gleitzer, C.; Aubry, J. Préparation et Propriétés d'un Oxyde de Lithium-Cuivre(III): Li_3CuO_3. *Revue de Chimie Minerale* **1975**, *12* (3), 203–209.

(8) Berger, R.; Önnerud, P.; Laligant, Y.; Le Bail, A. The Structure of $Li_3Cu_2O_4$, a Compound with Formal Mixed Valence. *Journal of Alloys and Compounds* **1993**, *190* (2), 295–299.

(9) Greaves, C.; Katib, S. M. A. The Structures of Li_5BiO_5 and Li_5SbO_5 from Powder Neutron Diffraction. *Materials Research Bulletin* **1989**, *24* (8), 973–980.

(10) Hauck, J.; Hirschberg, A. Über Eine Hochdruckmodifikation Des Li_6TeO_6. *Zeitschrift für Naturforschung B* **1969**, *24* (12), 1656–1656.

(11) Abrahams, I.; Lightfoot, P.; Bruce, P. G. $Li_6Zr_2O_7$, a New Anion Vacancy Ccp Based Structure, Determined by Ab Initio Powder Diffraction Methods. *Journal of Solid State Chemistry* **1993**, *104* (2), 397–403.

(12) Hodges, J. P.; Short, S.; Jorgensen, J. D.; Xiong, X.; Dabrowski, B.; Mini, S. M.; Kimball, C. W. Evolution of Oxygen-Vacancy Ordered Crystal Structures in the Perovskite Series $Sr_nFe_nO_{3n-1}$ (N=2, 4, 8, and ∞), and the Relationship to Electronic and Magnetic Properties. *Journal of Solid State Chemistry* **2000**, *151* (2), 190–209.
